# Genomic Diversity in the Endosymbiotic Bacterium *Rhizobium leguminosarum*

**DOI:** 10.3390/genes9020060

**Published:** 2018-01-24

**Authors:** Carmen Sánchez-Cañizares, Beatriz Jorrín, David Durán, Suvarna Nadendla, Marta Albareda, Laura Rubio-Sanz, Mónica Lanza, Manuel González-Guerrero, Rosa Isabel Prieto, Belén Brito, Michelle G. Giglio, Luis Rey, Tomás Ruiz-Argüeso, José M. Palacios, Juan Imperial

**Affiliations:** 1 Centro de Biotecnología y Genómica de Plantas, Universidad Politécnica de Madrid (UPM)—Instituto Nacional de Investigación y Tecnología Agraria y Alimentaria (INIA), Campus de Montegancedo UPM, 28223 Madrid, Spain; carmen.sanchez-canizares@plants.ox.ac.uk (C.S.-C.); beatriz.jorrin@plants.ox.ac.uk (B.J.); david.duran@uam.es (D.D.); marta.albareda@upm.es (M.A.); laura.rubio@upm.es (L.R.-S.); monica.lanza@upm.es (M.L.); manuel.gonzalez@upm.es (M.G.-G.); rosabelprieto@yahoo.es (R.I.P.); belen.brito@upm.es (B.B.); luis.rey@upm.es (L.R.); 2 Departamento de Biotecnología-Biología Vegetal, Escuela Técnica Superior de Ingeniería Agronómica, Alimentaría y de Biosistemas, Universidad Politécnica de Madrid (UPM), 28040 Madrid, Spain; 3 Department of Plant Sciences, University of Oxford, South Parks Road, OX1 3RB Oxford, UK; 4 Departamento de Biología, Facultad de Ciencias, Universidad Autónoma de Madrid (UAM), Ciudad Universitaria de Cantoblanco, Calle Francisco Tomás y Valiente 7, 28049 Madrid, Spain; 5 Institute for Genome Sciences (IGS), University of Maryland School of Medicine, Baltimore, MD 21201, USA; snadendla@som.umaryland.edu (S.N.); mgiglio@som.umaryland.edu (M.G.G.); 6 Instituto de Ciencias Agrarias, Consejo Superior de Investigaciones Científicas (CSIC), Serrano 115 bis, 28006 Madrid, Spain

**Keywords:** *Rhizobium leguminosarum*, nitrogen fixation, symbiosis, genome, plasmid

## Abstract

*Rhizobium leguminosarum* bv. *viciae* is a soil α-proteobacterium that establishes a diazotrophic symbiosis with different legumes of the *Fabeae* tribe. The number of genome sequences from rhizobial strains available in public databases is constantly increasing, although complete, fully annotated genome structures from rhizobial genomes are scarce. In this work, we report and analyse the complete genome of *R. leguminosarum* bv. *viciae* UPM791. Whole genome sequencing can provide new insights into the genetic features contributing to symbiotically relevant processes such as bacterial adaptation to the rhizosphere, mechanisms for efficient competition with other bacteria, and the ability to establish a complex signalling dialogue with legumes, to enter the root without triggering plant defenses, and, ultimately, to fix nitrogen within the host. Comparison of the complete genome sequences of two strains of *R. leguminosarum* bv. *viciae*, 3841 and UPM791, highlights the existence of different symbiotic plasmids and a common core chromosome. Specific genomic traits, such as plasmid content or a distinctive regulation, define differential physiological capabilities of these endosymbionts. Among them, strain UPM791 presents unique adaptations for recycling the hydrogen generated in the nitrogen fixation process.

## 1. Introduction

Rhizobia are applied across 400 million ha of agricultural land per annum to improve legume forage and crop production through symbiotic nitrogen fixation [1]. These bacteria must be able to survive in diverse soil environments and take advantage of ecological niches offered by the roots of many different plants [2]. In view of the importance of the symbiosis in the global nitrogen cycle, a very large number of rhizobial genomes have been sequenced (Genomes OnLine Database, [3]). However very few of those have been fully closed and carefully annotated. As a result, detailed studies on their genomic diversity are scarce. *Rhizobium leguminosarum* (*Rl*) includes biovars *viciae*, *trifolii*, and *phaseoli*, whose American type I strains were reclassified to a new taxon as *Rhizobium etli* [4]. Only four genome sequences of *Rl* strains have been published as complete genomes to date: *Rl* bv. *viciae* (*Rlv*) 3841, nodulating pea (*Pisum sativum*), lentil (*Lens culinaris*) and vetch (*Vicia* spp.) [5,6], and three of *Rl* bv. *trifolii* (*Rlt*): WSM1325, WSM2304, and WSM1689, clover symbionts (*Trifolium* spp.) [1,7,8].

Finished rhizobial genomes range in size from 6.53 Mb for *R. etli* CFN42^T^ up to 9.1 Mb for *Bradyrhizobium diazoefficiens* USDA110^T^. Despite their differences in host specificity, members of the genus *Rhizobium* characteristically contain a multipartite genome with several large plasmids (from one to six) that harbour genes for antibiotic resistance, symbiotic properties, or specific metabolic abilities, conferring other adaptive properties that allow these bacteria to grow under different conditions [9]. The number and sizes of plasmids vary among isolates, together with their GC content, which tends to be lower than that for the chromosome. This reduction in GC content reflects their external origin and the incidence of mobile genetic elements [9,10]. In the case of *Rlv* 3841, its genome size is 7.75 Mb [6], distributed in a circular chromosome of 5.05 Mb, and six plasmids: pRL12 (870 kb), pRL11 (684 kb), pRL10 (488 kb), pRL9 (352 kb), pRL8 (147 kb), and pRL7 (151 kb). All these plasmids harbour a *repABC* replication system, which is the most common system for plasmid replication in α-proteobacteria [11]. Among these replicons, core functions are clustered in the chromosome, whereas the accessory (adaptive) component of the genome is mainly encoded in the plasmids. Among these plasmids, pRL10 is the symbiotic plasmid, and plasmids pRL7 and pRL8 are mobilisable [6]. Strains *Rlt* WSM1325, WSM2304, and WSM1689 have genome sizes of 7.41, 6.87, and 6.90 Mb, harbouring five, four, and five plasmids, respectively [1,7,8]. The multipartite genome organization likely reflects the necessity for a large inventory of genes to maximize growth and survival in the complex environment of the soil, together with the additional genetic requirements for bacteroid physiology imposed by the plant host environment [12]. Plant-associated *Rhizobiales* have larger genomes than those from non-plant associated *Rhizobiales*, with an average of 6.81 Mb vs. 4.47 Mb, respectively [13]. Broad genomic studies with draft genome assemblies of multiple rhizobial strains have been undertaken [14,15,16]. Such studies could help to define what has been termed as “symbiome”, that is, the essential genes required by all rhizobia for nodulation and nitrogen fixation [14]. However, the conclusion that emerges from these reports is that the symbiome concept is still difficult to define. It seems that there is not a single core symbiome, what might reflect the different strategies that rhizobia have developed to successfully interact with their corresponding legume hosts. Thus, the availability of rhizobial complete and fully annotated genome sequences is a valuable tool to refine this analysis and further understand such strategies.

To this aim, we have sequenced, closed, and annotated the genome of *Rlv* UPM791. This genome has been analysed regarding its symbiotic and adaptive functions in comparison to other *R. leguminosarum* strains, focusing specifically on *Rlv* 3841 in view of its relevance as a model organism in *Rhizobium* research and because it is able to nodulate the same range of legumes. Results from this comparison revealed the large extent of adaptive genomic diversity between otherwise similar bacteria and emphasize its importance within this bacterial group.

## 2. Materials and Methods 

### 2.1. Growth Conditions & DNA Preparation

*Rlv* UPM791 is a spontaneous streptomycin-resistant derivative [17] of strain 128C53, a hydrogenase-positive [18] isolate from pea nodules received from J. Burton (Nitragin Co., Milwaukee, WI, USA). It was grown in tryptone yeast (TY) broth [19] at 28 °C during two days to carry out genomic DNA extraction. For 454 (Roche GS FLX, Roche, Basel, Switzerland) and Illumina (San Diego, CA, USA) platform sequencing, a Cetyl Trimethyl Ammonium Bromide (CTAB)-based protocol [20] was used, whereas for PacBio platform sequencing, DNA was purified with a DNA extraction blood tissue kit (Qiagen Hilden, Germany). DNA samples were quantified by Qubit fluorometry (Life Technologies, Carlsbad, CA, USA), and by Nanodrop spectrophotometry (Thermo Scientific, Waltham, MA, USA). 

### 2.2. Genome Sequencing, Assembly, & Quality Check

A 4 kb shotgun library was used for whole-genome sequencing by means of the 454 GS-FLX pyrosequencing technology (Roche, Branford, CT, USA) at the Institute for Genome Sciences (University of Maryland, Baltimore, MD, USA), from which 778,332 paired-end reads were obtained, covering 20× of the genome. PacBio RNA-Seq II (RSII) sequencing (2 Single Molecule Real Time (SMRT) cells) was carried out at the University of Washington-Seattle (Laboratory of Biotechnology and Bioanalysis, WA, USA). A total of 18,805 filtered reads were obtained, with a mean length of 8.8 kb and a mean coverage of 23×. An Illumina MiSeq v 2.0 (2 x PE200) shotgun library was constructed and sequenced, which generated 2,124,731 paired-end reads with a mean coverage of 117×. For assembly, the Hierarchical Genome Assembly Process (HGAP, [21]) was first used to assemble PacBio reads. The workflow consists of a quality trimming, assembly by overlap-layout-consensus, and a consensus polishing using Quiver (Pacific Biosciences, Menlo Park, CA, USA). Where necessary, Illumina reads and 454 assemblies (SPAdes v.3, [22], Celera Assembler CA5.4 [23]) were used to confirm the PacBio assemblies. The sequence of the smallest plasmid was assembled with MIRA v4.0.2 [24] using both PacBio and Illumina reads. Contigs were ordered and joined using Abacas v1.3.1 [25] and GAP4 [26] included in the Staden package [27]. Finally, Circlator v1.0.0 [28] was used to circularize sequences, to fix the origin of replication (*dnaA* in the chromosome and *repA* in plasmids), and to generate the Genome Atlas. The package AMOS v3.1.0 [29] was used to circularize the smallest plasmid. The presence of single nucleotide polymorphisms (SNPs), insertions, and deletions was checked using the same bioinformatics pipeline described by Jorrin & Imperial [30]. Briefly, using the assembled, closed genome, reads were aligned using Bowtie2 v2.3.0. [31], the output was transformed with Samtools v1.4. [32], and SNPs, insertions, and deletions were called using VarScan 2 v2.3.9. [33]. The alignment itself was visualized with IGV v2.3.92. [34]. SNPs, insertions, and deletions present in both Illumina sets (in >60% of the reads) were changed manually in Geneious v10.0.9 (Biomatters, Auckland, New Zealand).

### 2.3. Genome Annotation

Annotation of the genome was carried out using the Analysis Engine prokaryotic annotation pipeline from the Institute for Genome Sciences, University of Maryland School of Medicine [35] (http://ae.igs.umaryland.edu/cgi/index.cgi). Genes were predicted using Glimmer [36], tRNAscan-SE [37], and RNAmmer [38], with manual curation. The pipeline produces functional annotation from a variety of evidence sources, including: BER (BLAST_extend_repraze) pairwise matches, hidden Markov model (HMM), transmembrane HMM (TMHMM), SignalP, and PROSITE matches. Several types of annotation are attached to each protein based on the available evidence, including: Enzyme Commission (EC) numbers, Gene Ontology (GO) terms, TIGRfams, and protein names. 

### 2.4. Comparative Genomics

#### 2.4.1. 16S Phylogeny and Average Nucleotide Identity

16S ribosomal RNA (rRNA) nucleotide and *repABC* concatenated amino acid sequences were downloaded from JGI-IMG (Joint Genome Institute-Integrated Microbial Genomes & Microbiomes). Sequences were aligned and phylogenetically analysed using MEGA 6 [39]. Average Nucleotide Identity (ANI) using MUMmer [40] as the alignment algorithm (ANIm) was calculated using the JSpecies package [41]. Distances were calculated as 1-ANIm and trees were computed with the UPMGA [42] method using MEGA 6 package. In the case of the plasmid analysis, Average Nucleotide Identity using BLAST as the alignment algorithm (ANIb) was calculated using the web server JSpeciesWS [43]. 

#### 2.4.2. Protein Cluster Analysis 

CMGBiotools [44] was used to cluster proteins of *Rlv* UPM791 with those of *Rlt* WSM1325 (NC_012848, NC_012850, NC_012852-NC_012854, NC_012858, [7]), *Rlt* WSM1689 (CP007045-CP007050, [8]), *Rlt* WSM2304 (CP001191-CP001195, [1]), *Rlt* CB782 (CP007067-CP007070, [45]), and *Rlv* 3841 (NC_008378-NC_008384, [6]). 

## 3. Results

### 3.1. Rhizobium leguminosarum bv. viciae UPM791 Complete Genome Is a New Reference Sequence for Endosymbiotic Bacteria 

The genomic sequence of *Rlv* UPM791 was determined by means of a combination of 454 GLS-FLX pyrosequencing, Illumina, and PacBio technologies. Previous PacBio versions had been plagued by miscalled bases. However, recent improvements have dramatically increased its reliability [46], and we found this to be true for our sequences. After the recruitment of reads from two independent Illumina MiSeq sequence sets, only 30 SNPs were uncovered. One of them was localized in the chromosome and the remaining 29 in pRlvD, in a region between positions 60,000 and 88,000. In this region, 36 genes were predicted, out of which 16 open reading frames (ORFs) were annotated as transposase-related genes, one as integrase, and two as resolvases. It appears that regions with a high content of mobilisable elements contain an increased number of repeats and are, therefore, harder to assemble [47,48]. Seven insertions were also identified, all of them localized in the pRlvD plasmid, presumably for similar reasons. Additionally, 74 deletions were detected across the entire genome (32 in the chromosome, nine in pRlvA, four in pRlvB, 12 in pRlvE, six in pRlvC, and 11 in pRlvD). Sixty-six of those deletions consisted of the absence of a G or a C within either a rich GC-content region, or a short stretch containing more than three repeats of these nucleotides.

**Figure 1:**
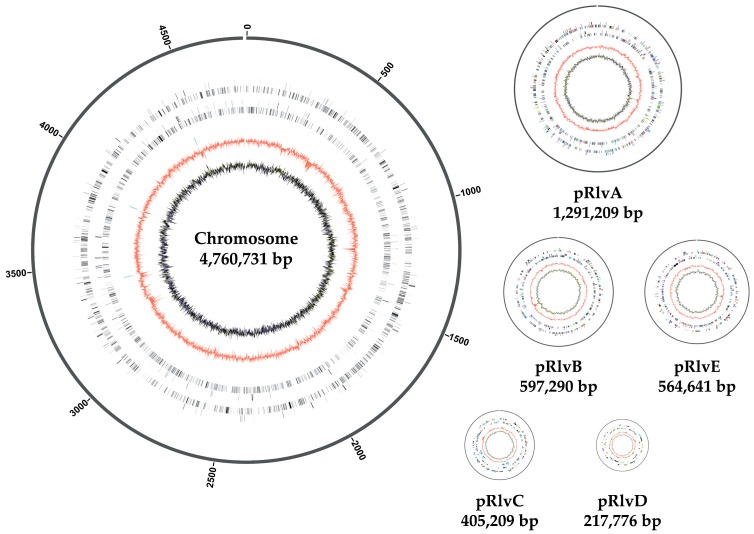
Genome Atlas of *R. leguminosarum* bv. *viciae*(*Rlv*) UPM791 chromosome and plasmid replicons at a relative scale. From the innermost circle: GCskew, followed by GCcontent, rRNAs, tRNAs, reverse CDS, and forward CDS. Names and sizes of the molecules are indicated.bp: Base pairs; CDS: Coding DNA sequence; rRNA: ribosomal RNA; tRNA: transfer RNA; *Rlv*: *Rhizobium leguminosarum* bv. *viciae*.

The *Rlv* UPM791 finished genome sequence (Figure 1) has a total size of 7,837,567 bp organized in a circular chromosome (4,760,731 bp, representing 61% of the total genome) and five extrachromosomal replicons, as follows: pRlvA (1,291,920 bp); pRlvB (597,290 bp); pRlvE (564,641 bp); pRlvC, the symbiotic plasmid (405,209 bp); and pRlvD (217,776 bp). A very similar codon usage pattern was observed in all the *Rlv* UPM791 replicons. GC content of the chromosome is 61%, whereas those of the plasmids are: 59.7% for pRlvA, 61% for pRlvB, 57.3% for pRlvC, 58.5% for pRlvD, and 60.9% for pRlvE. In all the replicons, the high GC content typical of *Rhizobiaceae* implies a high frequency of GC-rich codons. Consequently, amino acids corresponding to GC-containing codons are overrepresented.

The genome is predicted to encode 7380 genes, with 7318 coding DNA sequences (CDS), nine rRNA (clustered in three ribosomal operons), and 53 transfer RNA (tRNA) (Table 1). All the rRNA and tRNAs are found in the chromosome. All three rRNA clusters have an insertion of a tRNA-Met before the 5S gene and a tRNA-Ala and tRNA-Ile between the 23S and 16S genes. This genetic structure appears to be conserved across the rRNA operons of rhizobial genomes [49]. The general features and statistics of the *Rlv* UPM791 genome compared to those from reference genomes are presented in Table 1.

The 7318 CDS were classified into 19 different TIGRfam categories (each CDS could be classified into more than one category, see Supplementary Table S1). The majority of these genes (ca. 50%) are hypothetical proteins (19.66%), proteins of an unknown function (28.13%), and unclassified (9.07%), representing more than 56% of the total genome. A total of 4292 proteins received annotations other than “hypothetical protein” from the IGS (Institute for Genomic Sciences, Baltimore, MD, USA) prokaryotic annotation pipeline [25], with a differential distribution of categories among replicons. The chromosome harbours CDS with TIGRfam categories related to amino acid, protein, purines, pyrimidines, nucleosides, and nucleotides synthesis, as well as the cell envelope. The largest plasmid pRlvA contains the highest number of CDSs classified as regulatory functions. Plasmid pRlvB shows the highest number of CDSs allocated into the energy metabolism category and signal transduction. Plasmid pRlvE carries a high percentage of CDS categorized as fatty acids and transport and binding proteins. The symbiotic plasmid pRlvC shares with the chromosome the highest percentage of CDSs classified into DNA metabolism. This plasmid also has the largest percentages in terms of the biosynthesis of cofactors, prosthetic groups, and carriers, central intermediary metabolism, and protein fate. The smallest plasmid, pRlvD, holds the largest percentage of CDSs classified as cell processes and mobile and extrachromosomal elements (mainly transposons and integrases). 

Although only sequenced to a draft level, the genome sequence of *Rlv* UPM791 parent strain 128C53 is available in databanks (SAMN02441035) and is essentially identical, although in the particular isolate that was sequenced, the smallest plasmid pRlvD was missing. A known difference between both genomes should be related to the streptomycin-resistance mutation in *Rlv* UPM791. Many high-level streptomycin resistance (Str^R^) mutations map within the small ribosomal subunit protein S12 (*rpsL*) coding sequence [50]. A comparison of RpsL gene product sequences from *Rlv* UPM791 (RLV_4249) and 128C53 (WP_003547537.1) showed the presence of a K43R mutation in *Rlv* UPM791 RpsL, a common alteration in high-level Str^R^ mutants [51,52] and likely responsible for the Str^R^ phenotype.

### 3.2. Rhizobium leguminosarum bv. viciae UPM791 Global Genome Comparisons 

**Figure 2:**
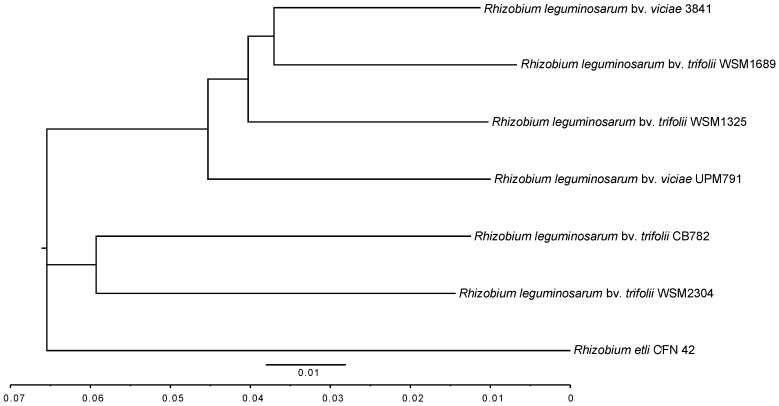
Average nucleotide identity MUMmer algorithm (ANIm) analysis. ANI-based UPMGA (unweighted pair group method with arithmetic mean) tree with the six representative *R. leguminosarum* genomes used in this study, using *Rhizobium etli* CFN42^T^ as the outgroup. The table below contains the ANIm values in percentage.

A comparison of the *Rlv* UPM791 16S rRNA gene sequence with other sequences in databanks placed this strain together with those from other *R. leguminosarum* strains (Supplementary Figure S1). For a more accurate analysis, the entire genome was aligned to the sequenced strains and the ANIm was also determined [41,53]. Our results indicate that *Rlv* UPM791 is phylogenetically related to *Rlt* WSM1325, *Rlv* 3841, and *Rlt* WSM1689 with 93.06, 93.05, and 92.50% sequence similarity, respectively (Figure 2). Considering that the ANI values between genomes of the same species are generally above 95%, these data would indicate that *Rlv* UPM791 should be considered a different genospecies. However, as reported by Kumar et al. [54], *R. leguminosarum* includes at least five distinct genospecies and could be considered a “species complex” rather than a regular species. An ANIm comparison of *Rlv* UPM791 with Kumar et al. genospecies A through E, suggests that *Rlv* UPM791 belongs to genospecies E (data not shown). 

**Figure 3:**
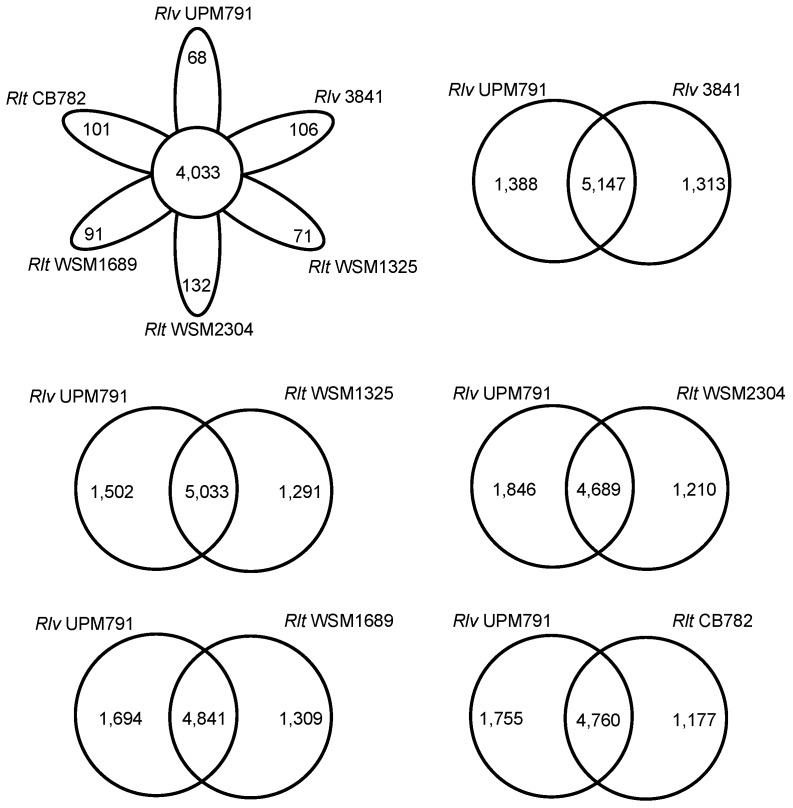
Venn diagram showing the number of protein families shared among strains *Rlv* UPM791, *Rlv* 3841, *Rlt* WSM2304, *Rlt* WSM1325, *Rlt* WSM1689, and *Rlt* CB782, and also between *Rlv* UPM791 and each of the other strains.

**Figure 4:**
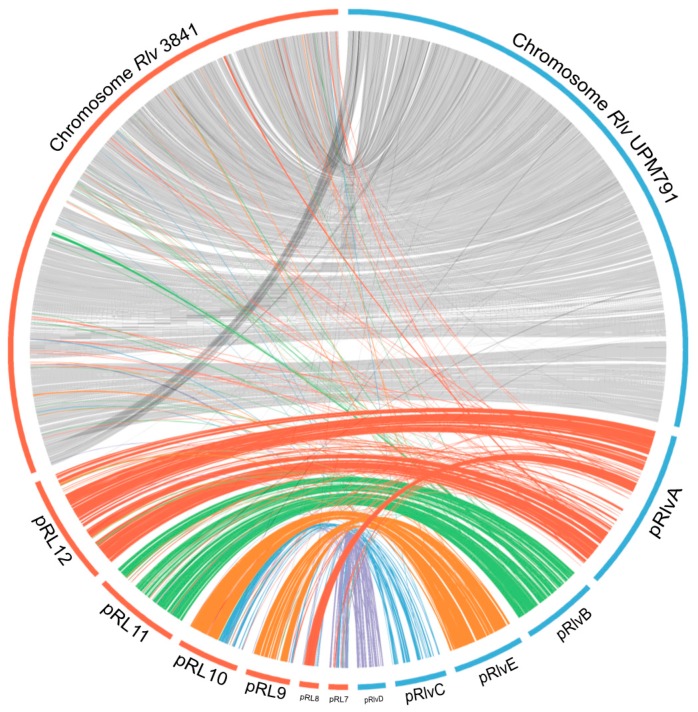
Circos representation of synteny between *Rlv* UPM791 and *Rlv* 3841 replicons.

A comparative protein cluster analysis of *Rlv* UPM791 was carried out using available, complete *R. leguminosarum* genome sequences: *Rlv* 3841, *Rlt* WSM1325, *Rlt* WSM2304, and *Rlt* WSM1689, as well as the high-quality permanent draft genome from *Rlt* CB782, an elite inoculant strain of commercial importance isolated from *Trifolium semipilosum* [45]. The six genomes shared 4033 clusters, with WSM2304 showing the highest number of exclusive CDS (132, Figure 3) and *Rlv* UPM791 the lowest (68). *Rlv* UPM791 and *Rlv* 3841 share the highest number of protein families, 5147. *Rlv* UPM791 also shares a high number of protein families with *Rlt* WSM1325 (5033). However, *Rlv* UPM791 shares the lowest number of protein families with *Rlt* WSM2304 (4689). In general, the chromosome is highly conserved in all the strains, as shown by the global synteny analysis in Supplementary Figure S2. When analysed in detail, four regions of the *Rlv* 3841 chromosome, of 52, 86, 99, and 48 kb, respectively, are not present in the *Rlv* UPM791 chromosome (Figure 4), with part of the fourth region appearing in *Rlv* UPM791 pRlvA.

### 3.3. High Diversity of the Plasmid-Associated Genome in Rhizobium leguminosarum

*Rlv* UPM791 extrachromosomal replicons have been named pRlvA, pRlvB, pRlvC, pRlvD, and pRlvE. All five plasmids have putative replication systems based on *repABC* genes. The largest extrachromosomal replicon is chromid pRlvA (1,291,920 bp and 1246 CDSs). Plasmids pRlvB and pRlvE have similar sizes, with respective values of 597,290 bp and 564,641 bp (588 and 545 CDSs, respectively). These two plasmids were previously visualized as a unique, more intense band in Eckhardt gels [55]. The two smallest plasmids are pRlvC (symbiotic plasmid, 405,209 bp and 366 CDSs) and pRlvD (217,776 bp, 239 CDSs). 

When the plasmid content was compared to that of *Rlv* 3841 (Figure 4, see Supplementary Figure S4 for more detail), we observed that four out of the five *Rlv* UPM791 plasmids had an equivalent in *Rlv* 3841 [6]. Plasmid pRlvA combines sequences from three different origins: (a) pRL12, the *Rlv* 3841 chromid [56]; (b) pRL8, a pea rhizosphere-specific plasmid with little collinearity (<5%) with other sequenced rhizobial genomes [57]; and (c) sequences not present in the *Rlv* 3841 genome, namely regions 342–426 kb (84 kb), 567–700 kb (133 kb), and 717–802 kb (85 kb). One of the noteworthy differences found between pRlvA and pRL12 is the absence of the Type VI Secretion System (T6SS), a system that is altogether missing in the *Rlv* UPM791 genome. With a GC content of 59.7%, pRlvA appears close to the chromosome (GC content of 61%), and would represent a megaplasmid or chromid, not uncommon in proteobacteria [10,56]. As such, pRlvA harbours some relevant genes, such as ATP-binding cassette (ABC) transporters (like the urea transport system UrtABCDE, RLV_0741 to RLV_0745), MCPs, or genes related to organic acids and sugar metabolism. 

pRlvB showed the highest degree of conservation with pRL11 (684,202 bp), its *Rlv* 3841 plasmid counterpart, as shown in more detail in Supplementary Figure S4. These plasmids exhibited an ANIb value of 92.76%, with a sequence coverage of 67.36% (Table 2). Interestingly, pRlvB harbours the only copy of the *minCDE* operon (RLV_1599-1601), involved in the accurate septum localization for cell division, implying that this replicon should be essential for the survival of this bacterium [58]. As in pRL11, pRlvB also includes the genes for cobalamin synthesis *cobFGHIJKLM* at the end of its sequence (RLV_1685-1678). Synteny analysis carried out with other *Rl* reference genomes suggests that large regions of this plasmid are also present in *Rlt* CB782 (plasmid 2), *Rlt* WSM1305 (pRL132502), *Rlt* WSM1689 (plasmid 2), and *Rlt* WSM2304 (pRLG202) (Supplementary Figure S2).

Plasmid pRlvE appears to be a cointegrate of sequences that in *Rlv* 3841 are found in plasmids pRL10 and pRL9, with 91.62 and 88.75% of the ANIb in the conserved parts, respectively (Table 2). Sequence conservation of pRlvE with plasmids pR132505 (294,782 bp) and pR132504 (350,312 bp), both from *Rlt* WSM1325 (90.97% and 88.80% ANIb, respectively), also emphasizes that plasmid pRlvE could be a cointegrate, with a total size (564,641 bp) roughly corresponding to the sum of plasmids pR132504 and pR132505. An alternative explanation would of course be that pRlvE has split into more than one plasmid in other *Rl* strains, as it occurs in *Rlv* 3841 and *Rlt* WSM1325.

Finally, the sequence of the smallest plasmid, pRlvD, was mapped against pRL7 (217,776 bp). However, contrary to the other replicons in *Rlv* UPM791, which shared a similar distribution of genes across functional categories, pRlvD consists mainly of transposons and insertion sequences. 

Surprisingly, while plasmids pRlvA, pRlvB, pRlvD, and pRlvE showed relatively good conservation with counterparts in *Rlv* 3841, symbiotic plasmid pRlvC exhibited a low degree of conservation with its *Rlv* 3841 counterpart, pRL10 (488,135 bp). This led us to analyse it in depth in the next section.

### 3.4. Rhizobium leguminosarum bv. viciae UPM791 Harbours a Unique Symbiotic Plasmid

**Figure 5:**
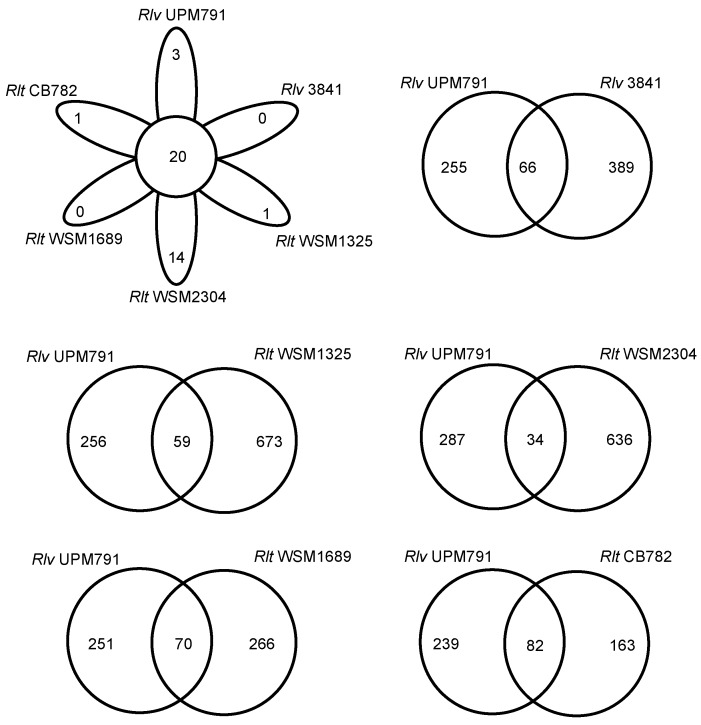
Venn diagram showing the number of protein families shared among the symbiotic plasmids from strains *Rlv* UPM791, *Rlv* 3841, *Rlt* WSM2304, *Rlt* WSM1325, *Rlt* WSM1689, and *Rlt* CB782, and also between that of *Rlv* UPM791 and those of each of the other strains.

**Figure 6:**
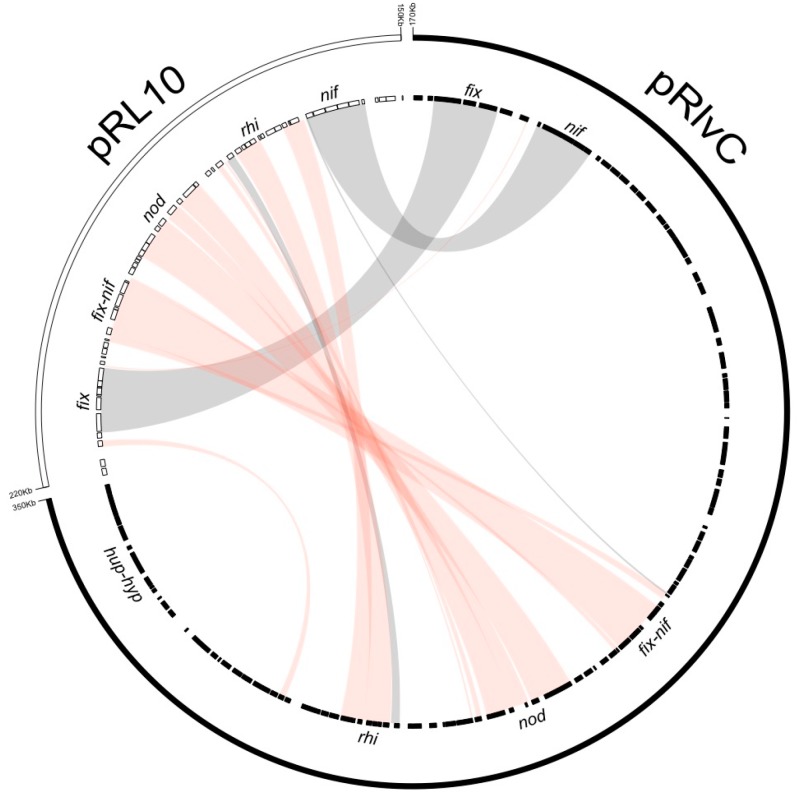
Circos representation of synteny between the symbiotic plasmids from *Rlv* UPM791 and *Rlv* 3841 strains.

The *Rlv* UPM791 symbiotic plasmid, pRlvC, was compared to each of the Sym plasmids of the reference genomes: *Rlv* 3841 pRL10, *Rlt* WSM1325 pR132501 (828,924 bp), *Rlt* WSM2304 pRLG201 (1,266,105 bp), *Rlt* WSM1689 Rleg3_Contig1811.4 (341,391 bp), and *Rlt* CB782 plasmid C219.4 (251,612 bp). When these plasmids were aligned using pRlvC as the reference sequence, a very low degree of conservation was observed. These results were consistent with those obtained from the comparison of the proteomes deduced from these symbiotic plasmids. A very low number of protein families were shared among the replicons (20, Figure 5). Symbiotic plasmids from *Rlv* UPM791 and *Rlt* CB782 strains share the highest number of protein families (82), despite being phylogenetically less related (Figure 5). Genes conserved among the five replicons are essentially restricted to those required to establish the symbiosis and fixing nitrogen (*nod*, *fix* and *nif* genes). As expected, and due to their differential host range, the genetic structure of the nodulation genes is only fully conserved between *Rlv* 3841 and *Rlv* UPM791, with small differences regarding those from the three *Rlt* strains. When the sequences of pRlvC and pRL10 were aligned, we observed that their arrangement was different (Figure 6). Whereas in pRL10, *fix* genes are followed by *nif*, *nod*, and *rhi* genes, in pRlvC, *fix* and *nif* genes are located in a different region, separated from the *rhi*, *nod*, and *fix-nif* clusters. pRlvC includes 13 genes involved in nodulation, *nodOTNMLEFDABCIJ*, located close to the rhizosphere-induced genes *rhiIABCR.* Genes required for nitrogenase comprise *nifHDKEN* and *nifAB*, and those also required for nitrogen fixation are *fixABCX* and *fixNOQPGHIS* (RLV_1890-RLV_1892 and RLV_1827-1834, respectively). 

In addition to the above genes, *Rlv* UPM791 symbiotic plasmid pRlvC also includes a large cluster of genes (*hupSLCDEFGHIJK hypABFCDEX*, corresponding to RLV_1962-1945), encoding the previously described NiFe hydrogenase system [59]. This hydrogenase enzyme recycles hydrogen generated in the nitrogenase catalytic cycle. Consistent with their symbiotic role, hydrogenase genes are co-expressed with *nif* genes via NifA-dependent regulation [60]. Interestingly, the *hup/hyp* gene cluster is highly conserved in a reduced number of *R. leguminosarum* strains, but absent in many others of the same species, including *Rlv* 3841 [61] and all *Rlt* strains analysed to date [62]. The *Rlv* UPM791 hydrogenase cluster contains *hupE*, encoding an energy-independent diffusion facilitator for the uptake of Ni(II) ions, required for synthesis of the hydrogenase heterobimetallic cofactor [63]. Similar *hup*/*hyp* clusters are present in some other species of *Rhizobium*, *Bradyrhizobium*, and *Azorhizobium* [64].

### 3.5. Specific Traits Define the Physiology of Each Rhizobial Strain

*Rlv* establishes a diazotrophic symbiosis with legumes of the tribe *Fabeae* (*Pisum*, *Lens*, *Vicia*, and *Lathyrus*) over a wide range of edaphoclimatic conditions. This broad host range could be partially explained by the existence of a large adaptive fraction of its genome that differs among isolates, as already suggested by the large number of plasmids. In the case of reference genomes studied in depth, such as *Ensifer meliloti* 1021 [65] or *Rlv* 3841 [6], these large genomes show a high number of genes devoted to solute uptake, the import and export of molecules, the production of cell-surface polysaccharides, regulatory functions, and also nitrogen metabolism [9], which could be necessary to fine tune required specificities.

#### 3.5.1. Metabolic Pathways 

The IGS prokaryotic annotation pipeline assigned 343 *Rlv* UPM791 proteins to the energy metabolism category and 88 proteins potentially involved in the synthesis of cell-surface polysaccharides and peptidoglycan. Metabolic pathways that are differently covered in *Rlv* UPM791 and *Rlv* 3841 are, for example, fluorobenzoate and dicloroethane degradation, with the BenAB (2-halobenzoate 1,2-dioxygenase, RLV_3415-3416) pathway absent in *Rlv* 3841. *Rlv* UPM791 also shows a higher number of proteins involved in beta-lactam resistance. Regarding specific traits, *Rlv* UPM791 has two copies of isocitrate dehydrogenase: the chromosomal RLV_4899 and RLV_1979, encoded in pRlvC and subjected to microoxic regulatory control (unpublished results); this second copy is absent in *Rlv* 3841. It appears to be the result of a duplication or insertion event, as it is located close to a transposable element. *Rlv* 3841 has two pathways for malate conversion to pyruvate: Dme and phosphoenolpyruvate (PEP) carboxykinase (PckA)/pyruvate kinase (PykA) [66]. These pathways are also present in *Rlv* UPM791: Dme, RLV_2776; and PckA, RLV_7044, with two copies of PykA: RLV_6265, similar to the protein of *Rlv* 3841, and RLV_3363, both in the chromosome. However, *Rlv* UPM791 contains a phosphoenolpyruvate carboxylase (PPC) that converts phosphoenolpyruvate to oxaloacetate by the addition of bicarbonate (HCO_3_^−^), thus feeding into the TCA cycle, which is absent in *Rlv* 3841. A unique characteristic in *Rlv* 3841 was the presence of two functional PHB synthases: a chromosomal one, active in free-living and undifferentiated bacteria (type I, PhaC1), and another in pRLl10, active in bacteroids (type III, PhaE PhaC2) [67]. However, *Rlv* UPM791 presents only the chromosomal type I PHB synthase equivalent to PhaC1, RLV_4485, but lacks the type III one, PhaE PhaC2, as most of the sequenced rhizobia do. This emphasizes once again the differences between the symbiotic plasmids of these endosymbiotic bacteria and their potential symbiotic behaviour. 

#### 3.5.2. Transport Systems

The high number of ABC transport systems harboured by rhizobia, in the order of *ca*. 200 (as compared to 57 for *Escherichia coli*), is particularly remarkable and a reflection of their lifestyle [68,69]. These transporters enable rhizobia to access a wide diversity of nutrients in the soil and the plant rhizosphere, even if they are at low concentrations [70]. This is the case, for example, for *Rlv* 3841, which harbours 183 complete ABC operons, representing 11% of its protein complement [6]. In *Rlv* UPM791, among those proteins with an assigned function, a large number of proteins are transport and binding proteins (906 proteins), the majority of which correspond to ABC transporters. For example, *Rlv* UPM791 has a full copy of both Aap and Bra (RLV_4520-4523, RLV_6024-6027), two broad-specificity amino acid ABC transporters necessary for an effective nitrogen fixation in the bacteroids of *Rlv* 3841 [71]. Active transport efflux pumps represent the largest category of metal resistance systems [72]. For example, the *Rl* DmeRF system is a metal-responsive efflux mechanism acting as a key element for metal homeostasis under free-living and symbiotic conditions [73]. This system, present in the chromosome of *Rlv* UPM791 (RLV_3758-3759), controls the concentration of Ni and Co, which can be toxic at moderate concentrations but are essential microelements for microbial nutrition that participate in a variety of cellular processes, such as hydrogenase biosynthesis [74].

#### 3.5.3. Motility and Chemotaxis Proteins 

The presence of methyl-accepting chemotaxis proteins (MCPs) in the *Rlv* UPM791 genome was also analysed. These proteins are required in order to sense chemical gradients and direct the movements of the bacterium towards different attractants by chemotaxis [75]. Their structural variability relies upon the molecules they detect, harbouring two different domains: a methyl-accepting chemotaxis protein signaling domain and a N-terminal ligand-binding region (LBR). Soil bacteria are metabolically versatile, prepared to sense and respond to a wide range of compounds [76]. In the *Rlv* UPM791 genome, we have identified 35 MCP genes, with 22 of them located in the chromosome. Similarly, high numbers of MCP proteins were detected in the other *Rl* strains analysed (28, 31, 25, and 32 MCPs for strains *Rlv* 3841, *Rlt* WSM1325, *Rlt* WSM1689, and *Rlt* WSM 2304, respectively). A high number of MCPs per genome correlates with lifestyles involving complex behaviour or interactions [77], as is the case for rhizobia. A phylogenetic tree including MCPs from the five *Rl* strains studied (Supplementary Figure S5) revealed that 21 MCP genes (16 chromosomal and five plasmid-borne) present in *Rlv* UPM791 have a close ortholog in all the other four strains, thus indicating the existence of a shared core of chemical sensing machinery in this species. On the other hand, two MCP genes (RLV_0526 and RLV_0630) were not closely related to MCPs from any of the other strains; interestingly, the genes for these “orphan” MCPs are encoded in pRlvA. This set of MCPs might give specific sensing capabilities to some of the strains within this species. 

#### 3.5.4. Cell-Surface Polysaccharide Production

Bacterial surface polysaccharides have several functions, such as nutrient gathering, environmental stresses protection or attachment, and biofilm formation, ensuring adaptation to changing environmental conditions. In rhizobia, acidic extracellular polysaccharides (EPS) are crucial in the initial bacterial invasion for the establishment of a successful symbiosis with legumes, especially for the formation of indeterminate nodules [78]. *Rl* strains produce a characteristic EPS skeleton formed by octasaccharide repeating units, with glucose as the dominant sugar component [79]. However, they differ in the sugar composition of their repeating units, the length of the side chain, and the pattern of non-sugar decoration (acetyl, pyruvyl, and hydroxybutanoyl residues) [78]. The genes directing the biosynthesis of exopolysaccharides (*exo/exs* or *pss* genes) are found in large clusters either on the chromosome or on the megaplasmids [78]. In *Rlv* UPM791 and 3841, these genes are found in the chromosome. Both strains harbour a copy of the *exoB* (RLV_6933) and *exoY* (RLV_6099) genes, responsible for the synthesis of sugar precursors and unit assembly, respectively. The overall genetic organization of *pss* genes is conserved in *Rlv* strains 3841 and UPM791. Both genomes harbour two copies of the *pssA* gene (RLV_6029, RLV_4118 in *Rlv* UPM791), which encodes a conserved protein involved in the first step of the EPS synthesis located in a single ORF at a long distance from the other *pss* genes (RLV_5910-5935). In both *Rlv* strains, the *pss* cluster contains the EPS modification gene *pssRMK* and the polymerization and translocation machinery encoded by *pssTNOP* and *pssL* genes. However, they differ in the number of glycosyl transferases, with *pssGH* genes absent in *Rlv* UPM791, as happens in *Rlt* TA1 [80]; this difference suggests that *Rlv* UPM791 produces an alternatively structured EPS. Apart from the *pss* genes, other genes encoding proteins essential for EPS synthesis are also present in this large cluster in both *Rlv* strains, as is the case of the *prsDE* genes (RLV_3415-3416). They encode two components of a type I protein secretion system, responsible for the secretion of a broad range of substrates, such as the nodulation protein NodD, the glycanase PlyA, or the rhizobial adhesion protein RapA. Consistently, *rapA* (RLV_3418) and *plyA* (RLV_3417) genes are part of the large polysaccharide gene cluster in both strains [80]. 

#### 3.5.5. Secretion Systems 

Interactions of bacteria with other organisms, prokaryotic or eukaryotic, rely on their ability to export proteins through dedicated protein secretion machineries. The general secretion (Sec) and twin-arginine (Tat) translocation pathways, responsible for the majority of protein export into the periplasm, were identified in the chromosome of *Rlv* UPM791. The Sec pathway primarily translocates proteins containing a signal peptide and in an unfolded state. The translocase comprises seven proteins, including an ATPase, SecA (RLV_6542); a chaperone, SecB (RLV_7013); a translocation channel, complex SecYEG (RLV_4274, RLV_4240, and RLV_4771); and two additional membrane proteins, SecDF, that promote the release of the mature protein into the periplasm (RLV_3073, RLV_4445). The Tat pathway secretes folded proteins with twin arginine signal peptides. In *Rlv* UPM791, the Tat system consists of three subunits encoded in the *tatABC* operon (RLV_4436-4438). This system was shown to be essential in this strain for symbiosis with peas [81]. The sequence identity of the genes involved in the Sec and Tat pathways compared to *Rlv* 3841 is above 95%. 

In rhizobia, type I, III, IV, and VI secretion systems have been shown to be involved in symbioses with legume hosts [82]. *Rlv* UPM791 presents only type I (T1SS) and IV (T4SS) systems. A complete T1SS is encoded in pRlvA (RLV_1006-1012) and consists of two permeases, a LacI-type and a TetR-type regulator, two Haemolysin secretion protein D (HlyD) proteins, and a transmembrane protein. *Rlv* UPM791 lacks a *tolC* gene, encoding a common outer membrane protein able to interact with T1SS. Other transmembrane HlyD type I secretion proteins are encoded by genes RLV_3630, RLV_5928, RLV_2681, and RLV_2693. Putative auto-aggregation protein type I substrates correspond to genes RLV_5342, RLV_5930, and RLV_2396. A Type II secretion system, which depends on the activity of the Sec and/or Tat pathways to deliver their substrate proteins into the periplasm, was not found in *Rlv* UPM791. A Type III secretion system (T3SS), responsible for producing nodulation outer proteins (Nops), was reported in *Mesorhizobium loti*, *B. diazoefficiens*, and *Ensifer fredii* NGR234 [83,84,85], but appeared to be absent in *R. leguminosarum* strains. Whereas a Type VI secretion system (T6SS) is present in pRL12 from *Rlv* 3841 [6], no T6SS has been found in the *Rlv* UPM791 genome. However, both *Rlv* UPM791 and *Rlv* 3841 contain a T4SS *tra/trb* conjugative system in their symbiotic plasmids. In pRlvC, this T4SS is adjacent to the *repABC* genes, a situation also found in pSyms from several *Rhizobium* strains, including pRL10 from *Rlv* 3841 [6], pRtrCIAT899a from *Rhizobium tropici* CIAT899, and pPRF81b from *Rhizobium* sp. PRF81 [86]. All these plasmids also contain *traI* and *traR*, suggesting a Quorum Sensing-dependent regulation. This system has also been located in the *M. loti* symbiosis island, indicating a potential for the transmission of this chromosomal island [83] that has been experimentally confirmed [87]. An additional Type-IV pilus T4SS cluster [88], containing putative genes virB1-B11 (RLV_0329-0340), was identified in pRlvA. Type-IV pili have been described as virulence factors in bacteria, and can be involved in the colonization of surfaces in different genera of Gram-negative bacteria [89]. A pilus-assembly system, similar to that of *Rlv* 3841, appears in the operon RLV_7221-7252. Finally, we found one type V autotransporter barrel domain protein (RLV_5683) in the *Rlv* UPM791 genome, highly similar to *Rlv* 3841 RL1196.

#### 3.5.6. Control of Microoxic Metabolism 

The *fixNOQP fixGHIS* operons encode a *cbb*_3_-type cytochrome oxidase that is induced under microoxic conditions and is essential for respiration during symbiotic nitrogen fixation. As mentioned above, these genes are present in the symbiotic pRlvC plasmid in *Rlv* UPM791 (RLV_1827-1830, RLV_1831-1834). The regulation of these operons in rhizobia has been thoroughly studied in *E. meliloti* and in *B. diazoefficiens*, where they are under the control of the oxygen sensor/regulator/regulator system FixLJK that also controls the expression of the key nitrogen fixation regulator NifA [90]. In *R. leguminosarum* and *R. etli*, a canonical FixLJK system is absent, and *fixNOQP fixGHIS* expression is controlled by FnrN [91,92,93,94,95]. As previously shown in our laboratory, in *Rlv* UPM791, NifA and FnrN constitute the main regulators of both nitrogenase and hydrogenase expression, thus ensuring their coordinated expression [94,96]. While *nifA* is present as a single copy gene (RLV_1894) located, as in *Rlv* 3841, in the symbiotic plasmid pRlvC, *fnrN* is present in two functional copies in *Rlv* UPM791, one in the chromosome (*fnrN1*, RLV_5077), and the other in pRlvC (*fnrN2*, RLV_1980). This sets *Rlv* UPM791 apart from *Rlv* 3841, which only contains a chromosomal copy [6], and confirms previous studies from our laboratory [92,93,94,96].

#### 3.5.7. Regulatory Proteins 

A total of 738 regulatory proteins were found in the *Rlv* UPM791 genome, constituting *ca*. 10% of the predicted proteins (data not shown). We made a specific search for transcriptional regulators of the LuxR-type family in the genome of *Rlv* UPM791. These proteins have two characteristic domains: an autoinducer-binding domain that binds AHLs in the N-terminal region, and the LuxR-family DNA-binding, helix-turn-helix (HTH) domain in the C-terminal region [97]. The latter domain is a general DNA-binding domain that is present in other, non-LuxR-like response regulators. We identified 21 candidate proteins from which only five also included an autoinducer-binding domain, indicating their potential role in Quorum Sensing (QS): RLV_5631 (243 aa), RLV_3298 (244 aa), RLV_1922 (247 aa), RLV_6914 (251 aa), and RLV_2364 (270 aa). Among these proteins we found the QS systems previously described in our laboratory [98]: CinR (RLV_5631), with its cognate synthase CinI (RLV_5632; 221 aa); and RhiR (RLV_1922), with its corresponding synthase RhiI (RLV_1926; 185 aa). Both systems are similar to their counterparts in *Rl* A34 and *Rlv* 3841 strains. In all these genomes, CinRI is located in the chromosome, whereas RhiRI is encoded in the symbiotic plasmid. For *Rlv* UPM791, Cantero (2005) [98] reported that the *Rlv* 3841 *raiRI* system was absent in this strain; our genome analysis suggests that the same applies for the LuxR orphan regulator *bisR* [99]. No other QS synthase genes were found associated with the remaining LuxR homologues, indicating that they are probably orphan LuxR proteins [100]. Not included among these five potential LuxR functional proteins is the pseudogene RLV_2010, which corresponds to TraR and that, as previously shown [98], presents a frameshift mutation located in the autoinducer binding domain. However, sequence analysis of the gene for the associated TraI synthase suggests that it is functional, although further studies are needed to confirm its potential role in the cell-cell communication machinery of *Rlv* UPM791. The presence of the antirepressor TraM [98] was also confirmed (RLV_2009, 78 aa). 

Regarding other regulatory proteins important in rhizobia, we found a high number of GntR-like regulators in *Rlv* UPM791 (50). Well-known members of the GntR family in rhizobia control diverse processes, such as rhizopine catabolism in *E. meliloti* (MocR, [101]) or arabinose metabolism in *B. diazoefficiens* (AraR, [102]). LysR-family transcriptional regulators were also represented, among them NodD (RLV_1905), an important LysR regulator in rhizobia that mediates the activation of *nod*-gene expression in response to plant-produced flavonoids [103]. Among the ArsR-type regulators, the *R. leguminosarum* transcriptional repressor NolR acts on *nod*-gene expression after induction by NodD, which is required for optimal nodulation in some hosts [104], and is usually located in chromosomes or chromids [86]. In *Rlv* UPM791, it was accordingly found in the chromosome (RLV_5806), whereas a *nolR*-like gene is located in pRlvA (RLV_1120), a situation also found in *Rlv* 3841 (RL3537 and pRL120789). RosR is an additional transcriptional regulator involved in the positive regulation of exopolysaccharide synthesis [105] and the homologous gene is also present in the chromosome of *Rlv* UPM791 (RLV_3788). 

#### 3.5.8. Stress Proteins

Stress conditions are a key aspect in the definition of different symbiotic structures [106]. As in other host-microbe associations, endosymbiotic forms of rhizobia are subjected to different types of stress, such as the presence of antimicrobial NCR peptides [107]. Relevant members of the stress-response machinery are the small Heat-Shock Proteins (sHSPs), ubiquitous chaperones bearing a characteristic alpha-crystalline domain 80–100 residues long. These proteins are able to prevent the irreversible aggregation of proteins following denaturation due to high temperatures or other stress factors [108]. *Rlv* UPM791 encodes 7 sHSPs, five of which are located in plasmids (RLV_2751, RLV_6296, RLV_0361, RLV_0502, RLV_0817, RLV_0818, and RLV_1399). The genomes of *Rlv* 3841, *Rlt* WSM1325, and *Rlt* WSM1689 contain four, five, and five sHSPs, respectively. This is in sharp contrast to the situation commonly found in most bacteria, where no more than one member of the family is found per genome (71% of 318 genomes studied, [109]). In that study, a reduced group, including just 9% of the genomes studied, contained more than four sHSPs. This group included all legume endosymbiotic bacteria analysed, with the exception of *Rlv* 3841, which contains four sHSPs. It is tempting to speculate that a high number of sHSPs allows the rhizobia to cope with different kinds of stresses during their complex life style. A known source of stress for bacteria within indeterminate legume nodules is the presence of antimicrobial NCR (Nodule-specific Cysteine-Rich) peptides first described in alfalfa [107] and later on in other legumes showing differentiated bacteroids, including the pea [110]. NCR peptides are able to induce changes in free-living bacterial cells, such as the inhibition of cell division and endoreduplication, similar to those observed in bacteroids. Tolerance to these peptides under symbiotic conditions requires the presence of BacA, a protein proposed to function as a peptide transporter and required for bacteroid development in alfalfa and pea nodules [111,112]. The *Rlv* UPM791 genome encodes a BacA homologue (RLV_5830) highly conserved (96% identity) with those from the other *Rlv* and *Rlt* strains analyzed here. 

## 4. Discussion

Genomic approaches are being used nowadays to define and understand the genetic systems from bacterial symbionts implicated in the interaction with their respective hosts. The genome sequences of strains from model symbiotic rhizobial species, such as *E. meliloti* 1021 [65], *Rlv* 3841 [6], *R. etli* bv. *phaseoli* CFN42^T^ [113], *M. loti* MAFF303099 [83], or *Bradyrhizobium diazoefficiens* USDA110^T^ [84], have been determined, providing new insights into these symbiotic interactions. Moreover, with advances in sequencing technologies, a genomic encyclopaedia of root nodule bacteria (GEBA-RNB) was created by sequencing 107 genomes and capturing their phylogenetic and biogeographic diversity [45]. However, despite these holistic approaches, the number of complete rhizobial genomes still remains low. Given the wide genomic diversity of rhizobia uncovered by the GEBA-RNB study [45], this is a limitation to in-depth comparative studies.

One of the characteristics of rhizobial genomes is the presence of multiple plasmids, especially in fast-growing symbionts such as *Rlv* UPM791. In this strain, five different extra-chromosomal replicons were identified. These multipartite genomes are often associated with species that encounter a host, and may increase their adaptive potential by extending their metabolic and symbiotic capabilities [10,12]. For this reason, soil-dwelling species tend to have large genomes with extra-chromosomal DNA, allowing them to adjust to survival as free-living cells in the soil, or associated with their symbiotic partners [114]. Rhizobia appear to have evolved by means of horizontal gene transfer and gene duplication events [113]. This could explain why the plasmids from *Rlv* UPM791 contain large DNA regions also found in different plasmids harboured by other rhizobial strains, especially for pRlvA and pRlvE. The presence of IS elements and repeated DNA sequences facilitates co-integration events [115], and this might be the case for plasmids pRlvA and pRlvE. The co-integration of *E. meliloti* megaplasmids with the chromosome has been previously reported [116]. These phenomena have also been demonstrated in *E. fredii* NGR234 between the symbiotic plasmid, its chromosome, and even its megaplasmid [117]. 

As expected from previous studies [10,118,119], the chromosome displays a high synteny across different *Rl* genomes, whereas extra-chromosomal DNA represents an important source of variability among strains. The fact that all the strains used in the analysis share a core genome would explain why they are considered as the same species, whereas the adaptive genome is strain-specific. This is specifically the case for the symbiotic plasmid, which clusters genes needed for symbiosis in rhizobia [9]. Notably, this plasmid showed the lowest degree of sequence similarity across different *Rl* strains. However, when *Rlv* UPM791 pRlvC was compared to the symbiotic plasmids from the other four strains (*Rlv* 3841, *Rlt* WSM1325, *Rlt* WSM2304, *Rlt* WSM1689, and *Rlt* CB782), we observed that the regions harbouring the symbiotic gene clusters were very similar. For example, the 13 *nod* genes involved in nodulation showed the same structure in *Rlv* UPM791 and *Rlv* 3841, with the rhizosphere *rhiIABCR* genes [120] nearby this *nod* cluster along with the genes required for nitrogen fixation (*nif* genes). This fact probably indicates recent transfer events of symbiosis genes between distantly related plasmids, as was already proposed when comparing symbiotic plasmids pRL10 and pRL1 (a symbiotic plasmid from another *Rlv* isolate [121]). A similar situation was also observed in a study where the sequences of symbiotic plasmids from 14 different rhizobial strains were compared [14]. In that study, little identity was detected among them, despite having similar *nod*, *nif*, and *fix* gene clusters. This was also true when different genera and symbiotic strategies were compared, such as with the *M. loti* MAFF303099 and *E. meliloti* 1021 genomes; the *M. loti* symbiotic island contained no genes with orthologues in the *E. meliloti* genome, except for the highly conserved nodulation and nitrogen fixation genes [65]. In view of this conservation, it has been suggested that the symbiotic and nitrogen fixing abilities of these bacteria have been acquired through multiple lateral transfer events [14]. The extent of these events and the selective forces implicated in the evolution of the symbiotic traits have been thoroughly reviewed recently [122].

Rhizobial genomes appear to be highly dynamic, as reflected by the presence of a high number of insertion sequence (IS) elements and transposases [12]. In the *Rlv* UPM791 genome, this is clearly observed in the case of plasmid pRlvD, the counterpart of pRL7 from *Rlv* 3841, which contains an uncommon number of repeated sequences and mobile elements. This may be due to the accessory nature of many of the genes located in these replicons, which are thought to offer potential sites for recombination [115]. Plasmid pRlvD is very different from the rest of the *Rlv* UPM791 genome, a situation also noted for pRL7 [6], with more than 80% of foreign genes and/or genes of an unknown function and up to 31 pseudogenes. Among these genes, 28% encoded transposases [6]. 

Regarding large plasmids or megaplasmids, the term “chromid” was proposed to designate all those replicons that have a nucleotide composition and codon usage similar to those found in the chromosome, carry essential genes, but have a plasmid-type replication system and are larger than the accompanying plasmids [56]. These are characteristics that describe pRlvA, the largest *Rlv* UPM791 plasmid (1.3 Mb). Chromids appear to contain genus-specific genes and in *Rhizobium*, *Ensifer*, and *Agrobacterium*, it was already proposed that chromids were acquired from the common ancestor of these genera [123]. Their biological role appears to be that of enabling the bacterium to have a larger genome, allowing the chromosome to remain small, thus ensuring shorter generation times, a characteristic that might be desirable in fast-changing environments. This would be the case for fast-growing rhizobia, like *Rhizobium* and *Ensifer*, both harbouring chromids, whereas *Mesorhizobium* and *Bradyrhizobium*, which do not have such replicons, grow more slowly in free-living culture [56]. The difficulty in obtaining derivative strains cured of such chromids in the laboratory (our unpublished results for pRlvA) suggests their relevance for bacterial viability. 

Plasmid pRlvB, the *Rlv* UPM791 counterpart of pRL11 from *Rlv* 3841, could also be considered a chromid. These megaplasmids appear to be highly conserved among *Rl* strains, as was previously reported for pRL11, pRLG202 from *Rlt* WSM2304, pR132502 from *Rlt* WSM1325, and even p42e from *R. etli* CFN42^T^, thus suggesting that these plasmids might carry essential metabolic genes [14]. Megaplasmid p42e was considered to be a secondary chromosome in *R. etli*, as it is a highly stable replicon impossible to eliminate due to the presence of genes involved in primary metabolism [124]. One of the common characteristics of these highly conserved plasmids in rhizobia, also including pA from *R. etli* CIAT652, is the presence of the *minCDE* operon. The products of these genes are involved in the accurate localization of the cell division site, leading to septum formation [124]. This cluster is also present in the megaplasmid pNGR234b from *E. fredii* NGR234 [85] and in pRlvB, suggesting that this plasmid is also essential in *Rlv* UPM791. In a comprehensive review, DiCenzo and Finan (2017) [10] observed that megaplasmids are common in genera containing soil and marine bacteria interacting with eukaryotic hosts. They also noted that the presence of both a chromid and a megaplasmid in the genome seemed to be a unique trait, as only *ca*. 2.5% of the 1708 bacterial genomes examined had both replicons, uniquely widespread in the genus *Burkholderia* and the order Rhizobiales [10].

Rhizobia must survive within the legume nodule, but also in the heterogeneous environment of the soil, which might explain the high metabolic burden and versatility of their genomes. The accessory (adaptive) genes present in the rhizobial genomes sequenced to date might be related to their lifestyle, conferring adaptability to different ecological niches [6]. These bacteria are known to be rich in ABC transporters. While not reaching the 816 ABC genes identified in *Rlv* 3841, the *Rlv* UPM791 genome encoded 906 transport and binding proteins, thus predicting capabilities for a wide range of substrates to be taken up. These proteins appear to be widely distributed in *Rlv* 3841 genome, and are especially abundant on plasmids pRL12, pRL10, and pRL9. The number of predicted proteins with regulatory functions is also high (738 proteins), which is consistent with such large genomes, given that all the metabolic pathways should be tightly regulated [6]. Also high is the number of sHSPs in rhizobia, although the reasons for this are still unknown, as the exact function of these proteins has not been determined in rhizobia [109]. It is tempting to speculate that sHSPs might have a specific role in the stabilization of proteins partly denatured as a consequence of specific interactions with host factors. Interestingly, an sHSP from *Agrobacterium tumefaciens* has recently been implicated in virulence towards *Arabidopsis thaliana* through the protection of a component of the virulence (Vir) system [125]. 

Similar to what has been described in other rhizobia, *Rlv* UPM791 contains two gene clusters encoding chemotaxis systems (RLV_3079-3087 and RLV_6231-6241), orthologs of *che1* and *che2* systems, respectively, described in *Rlv* 3841 [126]. Although the multiplicity of chemotaxis systems is a common theme in plant-associated bacteria [127], there is still little information on the actual role that chemotaxis might play in symbiosis. Deletion of the *Rlv* 3841 *che1* system resulted in a significant reduction of nodulation competitiveness [126], likely due to less efficient localization on the root surface. In the case of *Rlv* UPM791, analysis of the genomic regions flanking *mcp* genes revealed that four out of the 35 chemoreceptor genes identified (RLV_3079, RLV_6234, RLV_6235, and RLV_6236) are physically linked to the chemotaxis-related operons. Interestingly, two *mcp* genes (RLV_5537 and RLV_6634) are linked to additional copies of *cheW*-like genes. CheW is known to participate in a highly ordered superlattice, also involving MCP chemoreceptors and CheA, a histidine kinase involved in signal transduction [128]. The physical association of *mcp* genes with *cheW* copies suggests that some MCPs might require specific mediators to attain optimal interaction with a CheA-dependent signaling pathway. The presence of multiple copies of *mcp* genes is widespread in plant-associated bacteria, likely providing additional tools to compete for root surface colonization [127]. However, information on the specific substrates recognized by rhizobial MCPs is also scarce. Work carried out with McpU, one of the most highly expressed MCPs in *E. meliloti*, has shown that this MCP binds proline, a major constituent of alfalfa seed exudate, and also directs chemotaxis towards other amino acids [129,130]. The existence of a significantly high number of *mcp* genes in *R. leguminosarum* (35 vs. 8 in *E*. *meliloti*) suggests a higher ability of this bacterium to direct the cell towards different compounds, perhaps linked to different specific hosts legumes. In addition, recent evidence indicates that, in bacteria with multiple *mcp* genes such as *Azospirillum* or *Myxococcus*, some chemoreceptors are involved in CheA- and CheW-dependent signal transduction pathways regulating other cellular functions such as the formation of resistant cyst cells, flocs, or biofilms [131]. It is possible that, also in rhizobia, some of the MCPs might be involved in other, non-chemotactic functions induced in response to specific chemical stimuli. 

Comparative genomics represents a valuable tool for capturing the specificities and generalities of each genome. When compared to its closest relative, *Rlv* UPM791 shows distinctive features that set it apart from *Rlv* 3841. For example, the pSym-borne *hup* system for hydrogen uptake is only present in *Rlv* UPM791, whereas a T6SS, absent in this strain, is present in pRL12 from *Rlv* 3841 [6]. The T6SS in *Rlv* 3841 could be non-functional, because it presents a natural deletion in the promoter region of the two main clusters. This deletion, together with the absence in *Rlv* UPM791, may indicate that T6SS is not necessary for symbiosis and that its presence could be deleterious, as it was shown in the ineffective symbiosis of *R. leguminosarum* RBL5523 with pea plants [132]. These *Rlv* strains also differ in the number of glycanases forming the exopolysaccharide, with *pssH* and *pssI* absent in *Rlv* UPM791, indicating the production of a different EPS. Another distinct feature was the presence of a second copy of FnrN in *Rlv* UPM791. Contrary to the situation in other rhizobia, FnrN controls both *Rlv* UPM791 hydrogenase and nitrogenase activities in the nodule, indicating the lack of a functional *fixK* gene in this strain [93]. A genomic FnrN^−^ mutant exhibited wild-type levels of hydrogenase activity [92], and only the *fnrN*1 *fnrN*2 double mutant formed ineffective nodules lacking both nitrogenase and hydrogenase activities [93]. The FnrN system has been hypothesized to be more flexible for adaptation to environments with fluctuating oxygen tensions [94], a situation probably encountered by the rhizobia, aerobic soil organisms that have to adapt to the microoxic conditions required inside the nodule to maintain an active nitrogenase enzyme. Moreover, a distinct redox environment has also been proposed for the rhizosphere after observing the induction of a rhizosphere-specific cytochrome oxidase, distinct from the purely aerobic *aa*_3_ cytochrome oxidase complex and from the high-affinity *cbb*_3_ cytochrome oxidase complex induced in the nodule, in *Rlv* 3841 [57]. 

Regarding the presence of intercellular communication systems in *Rlv* UPM791, both *cinRI* and *rhiRI* had been previously reported [98], as well as a *traI* synthase and a truncated *traR* regulator. The existence of other disrupted *traR* genes had already been reported, as is the case for *trlR* (*traR*-like regulator, [133]) in *A. tumefaciens* and *traR* from *R. tropici* CIAT899 [86]. In the case of *Rlv* UPM791 *traR*, a frameshift mutation has been found in the autoinducer binding domain. Conversely, the *trlR* mutation is located in the DNA-binding domain, which makes this protein still able to bind AHLs but prevents TraR activity in this bacterium, thus inhibiting conjugation [133]. In CIAT899, an insertion element is located upstream of the *traR* gene, disrupting its promoter [86]. The availability of the genome sequence allowed a global search for transcriptional regulators of the LuxR-type, and produced three additional proteins potentially involved in QS. Rhi and CinI genes seem to play an important role in the first steps of the symbiosis, as they were upregulated in the pea rhizosphere [57]. Further work is needed to ascertain the role of these proteins in *Rlv* UPM791. 

Despite all the above remarkable traits, a large fraction of the gene content is annotated as hypothetical genes or unknown function (25.2% for *Rlv* 3841 and 32.2% for *Rlv* UPM791), which calls for a large scale functional investigation to understand the overall capabilities of these genomes. High-throughput experimental approaches are being developed to decipher the role of these genes. Among them, the Insertion Sequencing (INSeq) transposon (Tn) mutagenesis-based approach appears to be a very powerful technique to study gene function at the genome scale [134,135,136,137]. INSeq has been adapted for use in the *Rhizobiaceae* and tested under different metabolic conditions in *Rlv* 3841 [138,139,140]. When different growth media were analysed, the group of genes encoding hypothetical proteins was one of the five major categories assigned to the core functional genome, highlighting the importance of these proteins in core cellular processes. INSeq was also very helpful in establishing plasmid essentiality in *Rlv* 3841, with pRL12 encoding enzymes predicted to function in central carbon metabolism, such as pRL120209 (putative *tpiA*) and pRL120210 (putative *rpiB*), thus explaining why pRL12 cured strains were unable to grow on minimal media [139,141]. INSeq also helped to understand how succinate catabolism is affected by low-O_2_ environments and how C_4_-dicarboxylic acids fuel N_2_ fixation [140]. In that study, *glnB* (RL2393), encoding the nitrogen regulatory protein PII, was identified as specifically essential for growth on succinate at 1% O_2_, a condition similar to that experienced by the bacteroid. These functional approaches would be useful, for example, to explain the reasons why *Rlv* UPM791 outcompetes *Rlv* 3841 when inoculated on pea plants [142] and to identify those genetic determinants involved in root infection and nodule occupancy.

## 5. Conclusions

The newly reported *Rhizobium leguminosarum* complete genome *Rlv* UPM791 and the genomic comparisons presented here advance our understanding of the symbiotic potential within this species and demonstrate the wide diversity of plasmids within the same species, especially for the symbiotic plasmid, the most unique replicon in each of the strains analysed. In common with all *Rhizobium leguminosarum* genomes analysed, *Rlv* UPM791 shows a high degree of metabolic versatility, enabling the species to survive in a wide range of environments and to adapt to changing lifestyles as free-living or symbiotic cells. However, pathways involved in hydrogen recycling or the regulation of nitrogen fixation are unevenly distributed amongst genomes, suggesting that *Rlv* UPM791 harbours key specific traits and can become a useful reference sequence for hydrogenase positive rhizobial strains.

## Figures and Tables

**Table:** 

	*Rlv* UPM791	*R. etli* CFN42^T^	*Rlv* 3841	*Rlt* CB782	*Rlt* WSM1325	*Rlt* WSM1689	*Rlt* WSM2304
*Rlv* UPM791							
*R. etli* CFN42^T^	87.89						
*Rlv* 3841	93.05	87.89					
*Rlt* CB782	89.16	88.15	89.14				
*Rlt* WSM1325	93.06	87.82	94.16	89.07			
*Rlt* WSM1689	92.50	87.85	94.38	89.06	93.56		
*Rlt* WSM2304	89.36	88.35	89.33	90.82	89.28	89.21	

**Table 1:** General features of *Rhizobium leguminosarum* (*Rl*) complete genome sequences (data from IMG/M database).

	*Rl viciae*	*Rl viciae*	*Rl trifolii*	*Rl trifolii*	*Rl trifolii*
	UPM791	3841	WSM1325	WSM2304	WSM1689
**Host plant**	pea, lentil, vetch, *Lathyrus*	pea, lentil, vetch, *Lathyrus*	clover	clover	clover
**Genome size (Mb)**	7.84	7.75	7.42	6.87	6.90
**GC content (%)**	60.51	60.86	60.77	61.18	60.94
**Ribosomal RNA operons**	3	3	3	3	3
**Transfer RNAs**	57	52	51	53	51
**Total protein-coding genes**	7318	7276	7232	6581	6709
**Plasmid no.**	5	6	5	4	5
**% genome in plasmids**	39	34.8	35.7	34	42.2
**Assembly No.**	PRJNA417467 *	ASM926v1	ASM2318v1	ASM2134v1	ASM51760v1
**Reference**	This work	[6]	[7]	[1]	[8]

* BioProject number.

**Table 2:** Average Nucleotide Identity based on BLAST algorithm (ANIb) for *Rlv* UPM791 replicons as compared to other *Rl* strains.

*Rlv* UPM791 Plasmids	ORFs Included	Strain and Plasmid	ANIb %	% Sequence Coverage
pRlvA	RLV_0001-RLV_1156	*Rlv* 3841 pRL12	90.44	43.03
		*Rlt* WSM1325 pRL132501	90.36	39.59
		*Rlt* WSM2304 pRLG201	83.02	33.75
pRlvB	RLV_1157-RLV_1685	*Rlv* 3841 pRL11	92.76	67.36
		*Rlt* WSM1325 pRL132502	91.89	63.57
		*Rlt* WSM2304 pRLG202	88.44	56.04
pRlvC	RLV_1686-RLV_2021	*Rlv* 3841 pRL10	88.59	20.41
pRlvD	RLV_2022-RLV_2227	*Rlv* 3841 pRL7	91.81	34.15
pRlvE	RLV_2228-RLV_2741	*Rlv* 3841 pRL10	91.62	40.42
		*Rlv* 3841 pRL9	88.75	37.44
		*Rlt* WSM1325 pRL132505	90.97	35.39
		*Rlt* WSM1325 pRL132504	88.80	37.39
		*Rlt* WSM2304 pRLG201	84.22	36.78
		*Rlt* WSM2304 pRLG204	86.98	33.77
Chr	RLV_2742- RLV_7380	*Rlv* 3841 chr	93.08	81.83
		*Rlt* WSM1325 chr	92.79	82.72
		*Rlt* WSM2304 chr	88.85	73.06

ORF: Open Reading Frame.

## Supplementary Materials

The following are available online at http://www.mdpi.com/2073-4425/9/2/60/s1, Figure S1. Maximum likelihood 16S rRNA phylogenetic tree; Figure S2. Global synteny between *Rlv* UPM791 and the *Rhizobium leguminosarum* complete genomes, including the chromosome and all the different plasmid replicons; Figure S3. NJ phylogenetic tree of RepABC concatenated protein sequences (numbers indicate the position of the genes according to the JGI database); Figure S4. *Rlv* UPM791 chromosome and plasmids mapped against *Rlv* 3841 replicons; Figure S5. NJ phylogenetic tree of MCP proteins from *R. leguminosarum* strains. Symbols indicate the strain of origin of each MCP as shown in the inset. Multiple alignment was carried out using CLC Main Workbench with the full sequences of a set of 152 MCP proteins. Phylogenetic tree was constructed using the Neighbor-Joining algorithm. Distance measures were calculated using the Kimura 2-parameter method with 1000 bootstrapped replicates; Table S1: List of TIGRfam categories for proteins found in the different replicons of *Rlv* UPM791 and in the overall genome (total).

## Data Deposition

The sequences of the strain reported in this paper have been deposited in the GenBank database (BioProject no. PRJNA417467).

## References

[1] Reeve W., O’Hara G., Chain P., Ardley J., Brau L., Nandesena K., Tiwari R., Malfatti S., Kiss H., Lapidus A. (2010). Complete genome sequence of *Rhizobium leguminosarum* bv. *trifolii* strain WSM2304, an effective microsymbiont of the South American clover *Trifolium polymorphum*. Stand. Genom. Sci..

[2] Downie J.A. (2010). The roles of extracellular proteins, polysaccharides and signals in the interactions of rhizobia with legume roots. FEMS Microbiol. Rev..

[3] Genomes OnLine Database. http://gold.jgi.doe.gov.

[4] Segovia L., Young J.P., Martinez-Romero E. (1993). Reclassification of American *Rhizobium leguminosarum* biovar *phaseoli* Type I strains as *Rhizobium etli* sp. nov.. Int. J. Syst. Bacteriol..

[5] Johnston A.W., Beringer J.E. (1975). Identification of the *Rhizobium* strains in pea root nodules using genetic markers. J. Gen. Microbiol..

[6] Young J.P., Crossman L.C., Johnston A.W., Thomson N.R., Ghazoui Z.F., Hull K.H., Wexler M., Curson A.R., Todd J.D., Poole P.S. (2006). The genome of *Rhizobium leguminosarum* has recognizable core and accessory components. Genome Biol..

[7] Reeve W., O’Hara G., Chain P., Ardley J., Brau L., Nandesena K., Tiwari R., Copeland A., Nolan M., Han C. (2010). Complete genome sequence of *Rhizobium leguminosarum* bv. *trifolii* strain WSM1325, an effective microsymbiont of annual mediterranean clovers. Stand. Genom. Sci..

[8] Terpolilli J., Rui T., Yates R., Howieson J., Poole P., Munk C., Tapia R., Han C., Markowitz V., Tatiparthi R. (2014). Genome sequence of *Rhizobium leguminosarum* bv *trifolii* strain WSM1689, the microsymbiont of the one flowered clover *Trifolium uniflorum*. Stand. Genom. Sci..

[9] Downie J.A., Young J.P. (2001). Genome sequencing. The ABC of symbiosis. Nature.

[10] diCenzo G.C., Finan T.M. (2017). The divided bacterial genome: Structure, function, and evolution. Microbiol. Mol. Biol. Rev..

[11] Cevallos M.A., Cervantes-Rivera R., Gutierrez-Rios R.M. (2008). The *repABC* plasmid family. Plasmid.

[12] MacLean A.M., Finan T.M., Sadowsky M.J. (2007). Genomes of the symbiotic nitrogen-fixing bacteria of legumes. Plant Physiol..

[13] Pini F., Galardini M., Bazzicalupo M., Mengoni A. (2011). Plant-bacteria association and symbiosis: Are there common genomic traits in alphaproteobacteria?. Genes.

[14] Black M., Moolhuijzen P., Chapman B., Barrero R., Howieson J., Hungria M., Bellgard M. (2012). The genetics of symbiotic nitrogen fixation: Comparative genomics of 14 rhizobia strains by resolution of protein clusters. Genes.

[15] Tian C.F., Zhou Y.J., Zhang Y.M., Li Q.Q., Zhang Y.Z., Li D.F., Wang S., Wang J., Gilbert L.B., Li Y.R. (2012). Comparative genomics of rhizobia nodulating soybean suggests extensive recruitment of lineage-specific genes in adaptations. Proc. Natl. Acad. Sci. USA.

[16] Sugawara M., Epstein B., Badgley B.D., Unno T., Xu L., Reese J., Gyaneshwar P., Denny R., Mudge J., Bharti A.K. (2013). Comparative genomics of the core and accessory genomes of 48 *Sinorhizobium* strains comprising five genospecies. Genome Biol..

[17] Leyva A., Palacios J.M., Mozo T., Ruiz-Argüeso T. (1987). Cloning and characterization of hydrogen uptake genes from *Rhizobium leguminosarum*. J. Bacteriol..

[18] Ruiz-Argüeso T., Hanus F.J., Evans H.J. (1978). Hydrogen production and uptake by pea nodules as affected by strains of *Rhizobium leguminosarum*. Arch. Microbiol..

[19] Beringer J.E. (1974). R factor transfer in *Rhizobium leguminosarum*. Microbiology.

[20] Wilson K. (2001). Preparation of genomic DNA from bacteria. Curr. Protoc. Mol. Biol..

[21] Chin C., Alexander D., Marks P., Klammer A.A., Drake J., Heiner C., Clum A., Copeland A., Huddleston J., Eichler E. (2013). Nonhybrid, finished microbial genome assemblies from long-read SMRT sequencing data. Nat. Methods.

[22] Bankevich A., Nurk S., Antipov D., Gurevich A.A., Dvorkin M., Kulikov A.S., Lesin V.M., Nikolenko S.I., Pham S., Prjibelski A.D. (2012). SPAdes: A new genome assembly algorithm and its applications to single-cell sequencing. J. Comput. Biol..

[23] Myers E.W., Sutton G.G., Delcher A.L., Dew I.M., Fasulo D.P., Flanigan M.J., Kravitz S.A., Mobarry C.M., Reinert K.H.J., Remington K.A. (2000). A whole-genome assembly of *Drosophila*. Science.

[24] Chevreux B., Wetter T., Suhai S. (1999). Genome sequence assembly using trace signals and additional sequence information. Computer Science and Biology: Proceedings of the German Conference on Bioinformatics, GCB’99, Hannover, Germany, 4–6 October 1999.

[25] Assefa S., Keane T.M., Otto T.D., Newbold C., Berriman M. (2009). ABACAS: Algorithm-based automatic contiguation of assembled sequences. Bioinformatics.

[26] Bonfield J.K., Smith K., Staden R. (1995). A new DNA sequence assembly program. Nucl. Acids Res..

[27] Staden R. (1996). The staden sequence analysis package. Mol. Biotechnol..

[28] Hunt M., Silva N.D., Otto T., Parkhill J., Keane J., Harris S. (2015). Circlator: Automated circularization of genome assemblies using long sequencing reads. Genome Biol..

[29] Treangen T., Sommer D., Angly F., Koren S., Pop M. (2011). Next generation sequence assembly with AMOS. Curr. Protoc. Bioinform..

[30] Jorrin B., Imperial J. (2015). Population genomics analysis of legume host preference for specific rhizobial genotypes in the *Rhizobium leguminosarum* bv. *viciae* symbioses. Mol. Plant-Microbe Interact..

[31] Langmead B., Salzberg S.L. (2012). Fast gapped-read alignment with Bowtie 2. Nat. Methods.

[32] Li H., Handsaker B., Wysoker A., Fennell T., Ruan J., Homer N., Marth G., Abecasis G., Durbin R., Genome Project Data P. (2009). The sequence Alignment/Map format and SAMtools. Bioinformatics.

[33] Koboldt D.C., Zhang Q.Y., Larson D.E., Shen D., McLellan M.D., Lin L., Miller C.A., Mardis E.R., Ding L., Wilson R.K. (2012). VarScan 2: Somatic mutation and copy number alteration discovery in cancer by exome sequencing. Genome Res..

[34] Robinson J.T., Thorvaldsdottir H., Winckler W., Guttman M., Lander E.S., Getz G., Mesirov J.P. (2011). Integrative genomics viewer. Nat. Biotech..

[35] Galens K., Orvis J., Daugherty S., Creasy H.H., Angiuoli S., White O., Wortman J., Mahurkar A., Giglio M.G. (2011). The IGS standard operating procedure for automated prokaryotic annotation. Stand. Genom. Sci..

[36] Delcher A.L., Harmon D., Kasif S., White O., Salzberg S.L. (1999). Improved microbial gene identification with GLIMMER. Nucl. Acids Res..

[37] Lowe T.M., Chan P.P. (2016). tRNAscan-SE On-line: Integrating search and context for analysis of transfer RNA genes. Nucleic Acids Res..

[38] Lagesen K., Hallin P., Rodland E.A., Staerfeldt H.H., Rognes T., Ussery D.W. (2007). RNAmmer: Consistent and rapid annotation of ribosomal RNA genes. Nucleic Acids Res,.

[39] Tamura K., Stecher G., Peterson D., Filipski A., Kumar S. (2013). MEGA6: Molecular evolutionary genetics analysis version 6.0. Mol. Biol. Evol..

[40] Delcher A.L., Salzberg S.L., Phillippy A.M. (2003). Using MUMmer to identify similar regions in large sequence sets. Curr Protoc Bioinformatics.

[41] Richter M., Rossello-Mora R. (2009). Shifting the genomic gold standard for the prokaryotic species definition. Proc. Natl. Acad. Sci. USA.

[42] Sneath P., Sokal R. (1973). Numerical Taxonomy: The Principles and Practice of Numerical Classification.

[43] Richter M., Rossello-Mora R., Oliver Glockner F., Peplies J. (2016). JSpeciesWS: A web server for prokaryotic species circumscription based on pairwise genome comparison. Bioinformatics.

[44] Vesth T., Lagesen K., Acar N., Ussery D. (2013). CMG-Biotools, a free workbench for basic comparative microbial genomics. PLoS ONE.

[45] Reeve W., Ardley J., Tian R., Eshragi L., Yoon J.W., Ngamwisetkun P., Seshadri R., Ivanova N.N., Kyrpides N.C. (2015). A genomic encyclopedia of the root nodule bacteria: Assessing genetic diversity through a systematic biogeographic survey. Stand. Genom. Sci..

[46] Rhoads A., Au K.F. (2015). PacBio sequencing and its applications. Genom. Proteom. Bioinform..

[47] Ricker N., Qian H., Fulthorpe R.R. (2012). The limitations of draft assemblies for understanding prokaryotic adaptation and evolution. Genomics.

[48] Loman N.J., Quick J., Simpson J.T. (2015). A complete bacterial genome assembled de novo using only nanopore sequencing data. Nat. Methods.

[49] Bautista-Zapanta J.N., Yoshida K., Suzuki K. (2005). Precise characterization of rDNA genes by intraspecies and inter-loci comparison of rdna sequences and biochemical analysis of ribosomal RNA molecules in *Agrobacterium tumefaciens*. Genes Genet. Syst..

[50] Carter A.P., Clemons W.M., Brodersen D.E., Morgan-Warren R.J., Wimberly B.T., Ramakrishnan V. (2000). Functional insights from the structure of the 30S ribosomal subunit and its interactions with antibiotics. Nature.

[51] Durfee T., Nelson R., Baldwin S., Plunkett G., Burland V., Mau B., Petrosino J.F., Qin X., Muzny D.M., Ayele M. (2008). The complete genome sequence of *Escherichia coli* DH10B: Insights into the biology of a laboratory workhorse. J. Bacteriol..

[52] Sreevatsan S., Pan X., Stockbauer K.E., Williams D.L., Kreiswirth B.N., Musser J.M. (1996). Characterization of *rpsL* and *rrs* mutations in streptomycin-resistant *Mycobacterium tuberculosis* isolates from diverse geographic localities. Antimicrob. Agents Chemother..

[53] Goris J., Konstantinidis K.T., Klappenbach J.A., Coenye T., Vandamme P., Tiedje J.M. (2007). DNA-DNA hybridization values and their relationship to whole-genome sequence similarities. Int. J. Syst. Evol. Microbiol..

[54] Kumar N., Lad G., Giuntini E., Kaye M.E., Udomwong P., Shamsani N.J., Young J.P.W., Bailly X. (2015). Bacterial genospecies that are not ecologically coherent: Population genomics of *Rhizobium leguminosarum*. Open Biol..

[55] Leyva A., Palacios J.M., Ruiz-Argueso T. (1987). Conserved plasmid hydrogen-uptake (*hup*)-specific sequences within Hup^+^
*Rhizobium leguminosarum* strains. Appl. Environ. Microbiol..

[56] Harrison P.W., Lower R.P., Kim N.K., Young J.P. (2010). Introducing the bacterial ‘chromid’: Not a chromosome, not a plasmid. Trends Microbiol..

[57] Ramachandran V.K., East A.K., Karunakaran R., Downie J.A., Poole P.S. (2011). Adaptation of *Rhizobium leguminosarum* to pea, alfalfa and sugar beet rhizospheres investigated by comparative transcriptomics. Genome Biol..

[58] De Boer P.A., Crossley R.E., Rothfield L.I. (1990). Central role for the *Escherichia coli minC* gene product in two different cell division-inhibition systems. Proc. Natl. Acad. Sci. USA.

[59] Palacios J.M., Manyani H., Martinez M., Ureta A.C., Brito B., Bascones E., Rey L., Imperial J., Ruiz-Argueso T. (2005). Genetics and biotechnology of the H_2_-uptake (NiFe) hydrogenase from *Rhizobium leguminosarum* bv. *viciae*, a legume endosymbiotic bacterium. Biochem. Soc. Trans..

[60] Ruiz-Argüeso T., Palacios J.M., Imperial J. (2001). Regulation of the hydrogenase system in *Rhizobium leguminosarum*. Plant Soil.

[61] Fernandez D., Toffanin A., Palacios J.M., Ruiz-Argueso T., Imperial J. (2005). Hydrogenase genes are uncommon and highly conserved in *Rhizobium leguminosarum* bv. *viciae*. FEMS Microbiol. Lett..

[62] Ruiz-Argueso T., Emerich D.W., Evans H.J. (1979). Hydrogenase system in legume nodules: A mechanism of providing nitrogenase with energy and protection from oxygen damage. Biochem. Biophys. Res. Commun..

[63] Albareda M., Rodrigue A., Brito B., Ruiz-Argueso T., Imperial J., Mandrand-Berthelot M.A., Palacios J. (2015). *Rhizobium leguminosarum* HupE is a highly-specific diffusion facilitator for nickel uptake. Metallomics.

[64] Baginsky C., Brito B., Imperial J., Palacios J.M., Ruiz-Argueso T. (2002). Diversity and evolution of hydrogenase systems in rhizobia. Appl. Environ. Microbiol..

[65] Galibert F., Finan T.M., Long S.R., Puhler A., Abola P., Ampe F., Barloy-Hubler F., Barnett M.J., Becker A., Boistard P. (2001). The composite genome of the legume symbiont *Sinorhizobium meliloti*. Science.

[66] Mulley G., Lopez-Gomez M., Zhang Y., Terpolilli J., Prell J., Finan T., Poole P. (2010). Pyruvate is synthesized by two pathways in pea bacteroids with different efficiencies for nitrogen fixation. J. Bacteriol..

[67] Terpolilli J.J., Masakapalli S.K., Karunakaran R., Webb I.U., Green R., Watmough N.J., Kruger N.J., Ratcliffe R.G., Poole P.S. (2016). Lipogenesis and redox balance in nitrogen-fixing pea bacteroids. J. Bacteriol..

[68] Mauchline T.H., Fowler J.E., East A.K., Sartor A.L., Zaheer R., Hosie A.H., Poole P.S., Finan T.M. (2006). Mapping the *Sinorhizobium meliloti* 1021 solute-binding protein-dependent transportome. Proc. Natl. Acad. Sci. USA.

[69] Linton K.J., Higgins C.F. (1998). The *Escherichia coli* ATP-binding cassette (ABC) proteins. Mol. Microbiol..

[70] Prell J., Poole P. (2006). Metabolic changes of rhizobia in legume nodules. Trends Microbiol..

[71] Prell J., White J.P., Bourdes A., Bunnewell S., Bongaerts R.J., Poole P.S. (2009). Legumes regulate *Rhizobium* bacteroid development and persistence by the supply of branched-chain amino acids. Proc. Natl. Acad. Sci. USA.

[72] Bruins M.R., Kapil S., Oehme F.W. (2000). Microbial resistance to metals in the environment. Ecotoxicol. Environ. Saf..

[73] Rubio-Sanz L., Prieto R.I., Imperial J., Palacios J.M., Brito B. (2013). Functional and expression analysis of the metal-inducible DmeRF system from *Rhizobium leguminosarum* bv. *viciae*. Appl. Environ. Microbiol..

[74] Brito B., Palacios J.M., Hidalgo E., Imperial J., Ruiz-Argueso T. (1994). Nickel availability to pea (*Pisum sativum* L.) plants limits hydrogenase activity of *Rhizobium leguminosarum* bv. *viciae* bacteroids by affecting the processing of the hydrogenase structural subunits. J. Bacteriol..

[75] Yost C.K., Rochepeau P., Hynes M.F. (1998). *Rhizobium leguminosarum* contains a group of genes that appear to code for Methyl-Accepting Chemotaxis proteins. Microbiology.

[76] Ortega A., Zhulin I.B., Krell T. (2017). Sensory repertoire of bacterial chemoreceptors. Microbiol. Mol. Biol. Rev..

[77] Lacal J., Garcia-Fontana C., Munoz-Martinez F., Ramos J.L., Krell T. (2010). Sensing of environmental signals: Classification of chemoreceptors according to the size of their ligand binding regions. Environ. Microbiol..

[78] Skorupska A., Janczarek M., Marczak M., Mazur A., Król J. (2006). Rhizobial exopolysaccharides: Genetic control and symbiotic functions. Microb. Cell Fact..

[79] Janczarek M., Urbanik-Sypniewska T. (2013). Expression of the *Rhizobium leguminosarum* bv. *trifolii pssA* gene, involved in exopolysaccharide synthesis, is regulated by RosR, phosphate, and the carbon source. J. Bacteriol..

[80] Janczarek M. (2011). Environmental signals and regulatory pathways that influence exopolysaccharide production in rhizobia. Int. J. Mol. Sci..

[81] Meloni S., Rey L., Sidler S., Imperial J., Ruiz-Argueso T., Palacios J.M. (2003). The Twin-Arginine Translocation (TAT) system is essential for *Rhizobium*-legume symbiosis. Mol. Microbiol..

[82] Nelson M.S., Sadowsky M.J. (2015). Secretion systems and signal exchange between nitrogen-fixing rhizobia and legumes. Front. Plant Sci..

[83] Kaneko T., Nakamura Y., Sato S., Asamizu E., Kato T., Sasamoto S., Watanabe A., Idesawa K., Ishikawa A., Kawashima K. (2000). Complete genome structure of the nitrogen-fixing symbiotic bacterium *Mesorhizobium loti*. DNA Res..

[84] Kaneko T., Nakamura Y., Sato S., Minamisawa K., Uchiumi T., Sasamoto S., Watanabe A., Idesawa K., Iriguchi M., Kawashima K. (2002). Complete genomic sequence of nitrogen-fixing symbiotic bacterium *Bradyrhizobium japonicum* USDA110. DNA Res..

[85] Schmeisser C., Liesegang H., Krysciak D., Bakkou N., Le Quere A., Wollherr A., Heinemeyer I., Morgenstern B., Pommerening-Roser A., Flores M. (2009). *Rhizobium* sp. strain NGR234 possesses a remarkable number of secretion systems. Appl. Environ. Microbiol..

[86] Ormeño-Orrillo E., Menna P., Almeida L.G., Ollero F.J., Nicolas M.F., Pains Rodrigues E., Shigueyoshi Nakatani A., Silva Batista J.S., Oliveira Chueire L.M., Souza R.C. (2012). Genomic basis of broad host range and environmental adaptability of *Rhizobium tropici* CIAT 899 and *Rhizobium* sp. PRF 81 which are used in inoculants for common bean (*Phaseolus vulgaris* L.). BMC Genom..

[87] Ramsay J.P., Sullivan J.T., Stuart G.S., Lamont I.L., Ronson C.W. (2006). Excision and transfer of the *Mesorhizobium*
*loti* R7A symbiosis island requires an integrase IntS, a novel recombination directionality factor RdfS, and a putative relaxase RlxS. Mol. Microbiol..

[88] Juhas M., Crook D.W., Hood D.W. (2008). Type IV secretion systems: Tools of bacterial horizontal gene transfer and virulence. Cell. Microbiol..

[89] Tomich M., Planet P.J., Figurski D.H. (2007). The tad locus: Postcards from the widespread colonization island. Nat. Rev. Microbiol..

[90] Dixon R., Kahn D. (2004). Genetic regulation of biological nitrogen fixation. Nat. Rev. Microbiol..

[91] Schluter A., Patschkowski T., Quandt J., Selinger L.B., Weidner S., Kramer M., Zhou L., Hynes M.F., Priefer U.B. (1997). Functional and regulatory analysis of the two copies of the *fixNOQP* operon of *Rhizobium leguminosarum* strain VF39. Mol. Plant-Microbe Interact..

[92] Hernando Y., Palacios J.M., Imperial J., Ruiz-Argueso T. (1995). The *hypBFCDE* operon from *Rhizobium leguminosarum* biovar *viciae* is expressed from an Fnr-type promoter that escapes mutagenesis of the FnrN gene. J. Bacteriol..

[93] Gutierrez D., Hernando Y., Palacios J.M., Imperial J., Ruiz-Argueso T. (1997). FnrN controls symbiotic nitrogen fixation and hydrogenase activities in *Rhizobium leguminosarum* biovar *viciae* UPM791. J. Bacteriol..

[94] Colombo M.V., Gutierrez D., Palacios J.M., Imperial J., Ruiz-Argueso T. (2000). A novel autoregulation mechanism of fnrn expression in *Rhizobium leguminosarum* bv. *viciae*. Mol. Microbiol..

[95] Lopez O., Morera C., Miranda-Rios J., Girard L., Romero D., Soberon M. (2001). Regulation of gene expression in response to oxygen in *Rhizobium etli*: Role of *fnrN* in *fixNOQP* expression and in symbiotic nitrogen fixation. J. Bacteriol..

[96] Brito B., Martinez M., Fernandez D., Rey L., Cabrera E., Palacios J.M., Imperial J., Ruiz-Argueso T. (1997). Hydrogenase genes from *Rhizobium leguminosarum* bv. *viciae* are controlled by the nitrogen fixation regulatory protein NifA. Proc. Natl. Acad. Sci. USA.

[97] Fuqua W.C., Winans S.C., Greenberg E.P. (1994). Quorum sensing in bacteria: The LuxR-LuxI family of cell density-responsive transcriptional regulators. J. Bacteriol..

[98] Cantero L. (2005). Análisis Genético y Proteómico de la Regulación de Los Sistemas de Autoinducción en *Rhizobium leguminosarum* bv. *viciae* UPM791 y 3841. Ph.D. Thesis.

[99] Frederix M., Downie A.J. (2011). Quorum sensing: Regulating the regulators. Adv. Microb. Physiol..

[100] Patankar A.V., Gonzalez J.E. (2009). Orphan LuxR regulators of quorum sensing. FEMS Microbiol. Rev..

[101] Rossbach S., Kulpa D.A., Rossbach U., de Bruijn F.J. (1994). Molecular and genetic characterization of the rhizopine catabolism (*mocABRC*) genes of *Rhizobium meliloti* L5-30. Mol. Gen. Genet..

[102] Pedrosa F.O., Zancan G.T. (1974). L-arabinose metabolism in *Rhizobium japonicum*. J. Bacteriol..

[103] Hungria M., Joseph C.M., Phillips D.A. (1991). *Rhizobium nod* gene inducers exuded naturally from roots of common bean (*Phaseolus vulgaris* L.). Plant Physiol..

[104] Kiss E., Mergaert P., Olàh B., Kereszt A., Staehelin C., Davies A.E., Downie J.A., Kondorosi A., Kondorosi E. (1998). Conservation of *nolR* in the *Sinorhizobium* and *Rhizobium* genera of the rhizobiaceae family. Mol. Plant-Microbe Interact..

[105] Janczarek M., Kutkowska J., Piersiak T., Skorupska A. (2010). *Rhizobium leguminosarum* bv. *trifolii* RosR is required for interaction with clover, biofilm formation and adaptation to the environment. BMC Microbiol..

[106] Schwartzman J.A., Ruby E.G. (2016). Stress as a normal cue in the symbiotic environment. Trends Microbiol..

[107] Kondorosi E., Mergaert P., Kereszt A. (2013). A paradigm for endosymbiotic life: Cell differentiation of *Rhizobium* bacteria provoked by host plant factors. Annu. Rev. Microbiol..

[108] Haslbeck M., Vierling E. (2015). A first line of stress defense: Small Heat Shock proteins and their function in protein homeostasis. J. Mol. Biol..

[109] Han M.-J., Yun H., Lee S.Y. (2008). Microbial Small Heat Shock proteins and their use in biotechnology. Biotechnol. Adv..

[110] Montiel J., Downie J.A., Farkas A., Bihari P., Herczeg R., Balint B., Mergaert P., Kereszt A., Kondorosi E. (2017). Morphotype of bacteroids in different legumes correlates with the number and type of symbiotic NCR peptides. Proc. Natl. Acad. Sci. USA.

[111] Haag A.F., Baloban M., Sani M., Kerscher B., Pierre O., Farkas A., Longhi R., Boncompagni E., Herouart D., Dall’Angelo S. (2011). Protection of *Sinorhizobium* against host cysteine-rich antimicrobial peptides is critical for symbiosis. PLoS Biol..

[112] Karunakaran R., Haag A.F., East A.K., Ramachandran V.K., Prell J., James E.K., Scocchi M., Ferguson G.P., Poole P.S. (2010). BacA is essential for bacteroid development in nodules of galegoid, but not phaseoloid, legumes. J. Bacteriol..

[113] Gonzalez V., Santamaria R.I., Bustos P., Hernandez-Gonzalez I., Medrano-Soto A., Moreno-Hagelsieb G., Janga S.C., Ramirez M.A., Jimenez-Jacinto V., Collado-Vides J. (2006). The partitioned *Rhizobium etli* genome: Genetic and metabolic redundancy in seven interacting replicons. Proc. Natl. Acad. Sci. USA.

[114] Bentley S.D., Parkhill J. (2004). Comparative genomic structure of prokaryotes. Annu. Rev. Genet..

[115] Mavingui P., Flores M., Guo X., Davila G., Perret X., Broughton W.J., Palacios R. (2002). Dynamics of genome architecture in *Rhizobium* sp. strain NGR234. J. Bacteriol..

[116] Guo X., Flores M., Mavingui P., Fuentes S.I., Hernandez G., Davila G., Palacios R. (2003). Natural genomic design in *Sinorhizobium meliloti:* Novel genomic architectures. Genome Res..

[117] Flores M., Mavingui P., Perret X., Broughton W.J., Romero D., Hernández G., Dávila G., Palacios R. (2000). Prediction, identification, and artificial selection of DNA rearrangements in *Rhizobium*: Toward a natural genomic design. Proc. Natl. Acad. Sci. USA.

[118] Krol J.E., Mazur A., Marczak M., Skorupska A. (2008). Application of physical and genetic map of *Rhizobium leguminosarum* bv. *trifolii* TA1 to comparison of three closely related rhizobial genomes. Mol. Genet. Genom..

[119] Crossman L.C., Castillo-Ramirez S., McAnnula C., Lozano L., Vernikos G.S., Acosta J.L., Ghazoui Z.F., Hernandez-Gonzalez I., Meakin G., Walker A.W. (2008). A common genomic framework for a diverse assembly of plasmids in the symbiotic nitrogen fixing bacteria. PLoS ONE.

[120] Cubo M.T., Economou A., Murphy G., Johnston A.W., Downie J.A. (1992). Molecular characterization and regulation of the rhizosphere-expressed genes *rhiABCR* that can influence nodulation by *Rhizobium leguminosarum* biovar *viciae*. J. Bacteriol..

[121] Rigottier-Gois L., Turner S.L., Young J.P.W., Amarger N. (1998). Distribution of *repC* plasmid-replication sequences among plasmids and isolates of *Rhizobium leguminosarum* bv. *viciae* from field populations. Microbiology.

[122] Remigi P., Zhu J., Young J.P.W., Masson-Boivin C. (2016). Symbiosis within symbiosis: Evolving nitrogen-fixing legume symbionts. Trends Microbiol..

[123] Slater S.C., Goldman B.S., Goodner B., Setubal J.C., Farrand S.K., Nester E.W., Burr T.J., Banta L., Dickerman A.W., Paulsen I. (2009). Genome sequences of three *Agrobacterium* biovars help elucidate the evolution of multichromosome genomes in bacteria. J. Bacteriol..

[124] Landeta C., Davalos A., Cevallos M.A., Geiger O., Brom S., Romero D. (2011). Plasmids with a chromosome-like role in rhizobia. J. Bacteriol..

[125] Hwang H.H., Liu Y.T., Huang S.C., Tung C.Y., Huang F.C., Tsai Y.L., Cheng T.F., Lai E.M. (2015). Overexpression of the *hspL* promotes *Agrobacterium tumefaciens* virulence in *Arabidopsis* under heat shock conditions. Phytopathology.

[126] Miller L.D., Yost C.K., Hynes M.F., Alexandre G. (2007). The major chemotaxis gene cluster of *Rhizobium leguminosarum* bv. *viciae* is essential for competitive nodulation. Mol. Microbiol..

[127] Scharf B.E., Hynes M.F., Alexandre G.M. (2016). Chemotaxis signaling systems in model beneficial plant-bacteria associations. Plant. Mol. Biol..

[128] Briegel A., Wong M.L., Hodges H.L., Oikonomou C.M., Piasta K.N., Harris M.J., Fowler D.J., Thompson L.K., Falke J.J., Kiessling L.L. (2014). New insights into bacterial chemoreceptor array structure and assembly from electron cryotomography. Biochemistry.

[129] Webb B.A., Hildreth S., Helm R.F., Scharf B.E. (2014). *Sinorhizobium meliloti* chemoreceptor McpU mediates chemotaxis toward host plant exudates through direct proline sensing. Appl. Environ. Microbiol..

[130] Webb B.A., Compton K.K., Del Campo J.S.M., Taylor D., Sobrado P., Scharf B.E. (2017). *Sinorhizobium meliloti* chemotaxis to multiple amino acids is mediated by the chemoreceptor McpU. Mol. Plant-Microbe Interact..

[131] He K., Bauer C.E. (2014). Chemosensory signaling systems that control bacterial survival. Trends Microbiol..

[132] Bladergroen M.R., Badelt K., Spaink H.P. (2003). Infection-blocking genes of a symbiotic *Rhizobium leguminosarum* strain that are involved in temperature-dependent protein secretion. Mol. Plant-Microbe Interact..

[133] Chai Y., Zhu J., Winans S.C. (2001). TrlR, a defective TraR-like protein of *Agrobacterium tumefaciens*, blocks TraR function in vitro by forming inactive TrlR:TraR dimers. Mol. Microbiol..

[134] Gawronski J.D., Wong S.M., Giannoukos G., Ward D.V., Akerley B.J. (2009). Tracking insertion mutants within libraries by deep sequencing and a genome-wide screen for *Haemophilus* genes required in the lung. Proc. Natl. Acad. Sci. USA.

[135] Goodman A.L., Wu M., Gordon J.I. (2011). Identifying microbial fitness determinants by insertion sequencing using genome-wide transposon mutant libraries. Nat. Protoc..

[136] Langridge G.C., Phan M.D., Turner D.J., Perkins T.T., Parts L., Haase J., Charles I., Maskell D.J., Peters S.E., Dougan G. (2009). Simultaneous assay of every *Salmonella typhi* gene using one million transposon mutants. Genome Res..

[137] Van Opijnen T., Bodi K.L., Camilli A. (2009). Tn-Seq: High-throughput parallel sequencing for fitness and genetic interaction studies in microorganisms. Nat. Methods.

[138] Perry B.J., Yost C.K. (2014). Construction of a mariner-based transposon vector for use in insertion sequence mutagenesis in selected members of the rhizobiaceae. BMC Microbiol..

[139] Perry B.J., Akter M.S., Yost C.K. (2016). The use of transposon insertion sequencing to interrogate the core functional genome of the legume symbiont *Rhizobium leguminosarum*. Front. Microbiol..

[140] Wheatley R.M., Ramachandran V.K., Geddes B.A., Perry B.J., Yost C.K., Poole P.S. (2017). Role of O_2_ in the growth of *Rhizobium leguminosarum* bv. *viciae* 3841 on glucose and succinate. J. Bacteriol..

[141] Hynes M.F., Quandt J., O’Connell M.P., Puhler A. (1989). Direct selection for curing and deletion of *Rhizobium* plasmids using transposons carrying the *Bacillus subtilis sacB* gene. Gene.

[142] Sanchez-Canizares C., Palacios J. (2013). Construction of a marker system for the evaluation of competitiveness for legume nodulation in *Rhizobium* strains. J. Microbiol. Methods.

