# Supplementary material for: Genomic Diversity in the Endosymbiotic Bacterium Rhizobium leguminosarum
Source: Genes (Basel). 2018 Jan 24;9(2):60. doi: 10.3390/genes9020060 (PMC5852556; doi:10.3390/genes9020060)

Figure S5. NJ phylogenetic tree of MCP proteins from *R. leguminosarum* strains. Symbols indicate the strain of origin of each MCP as shown in the inset. Multiple alignment was carried out using CLC Main Workbench with the full sequences of a set of 152 MCP proteins. Phylogenetic tree was constructed using Neighbor-Joining algorithm. Distance measures were calculated using Kimura 2-parameter method with 1000 bootstrapped replicates.

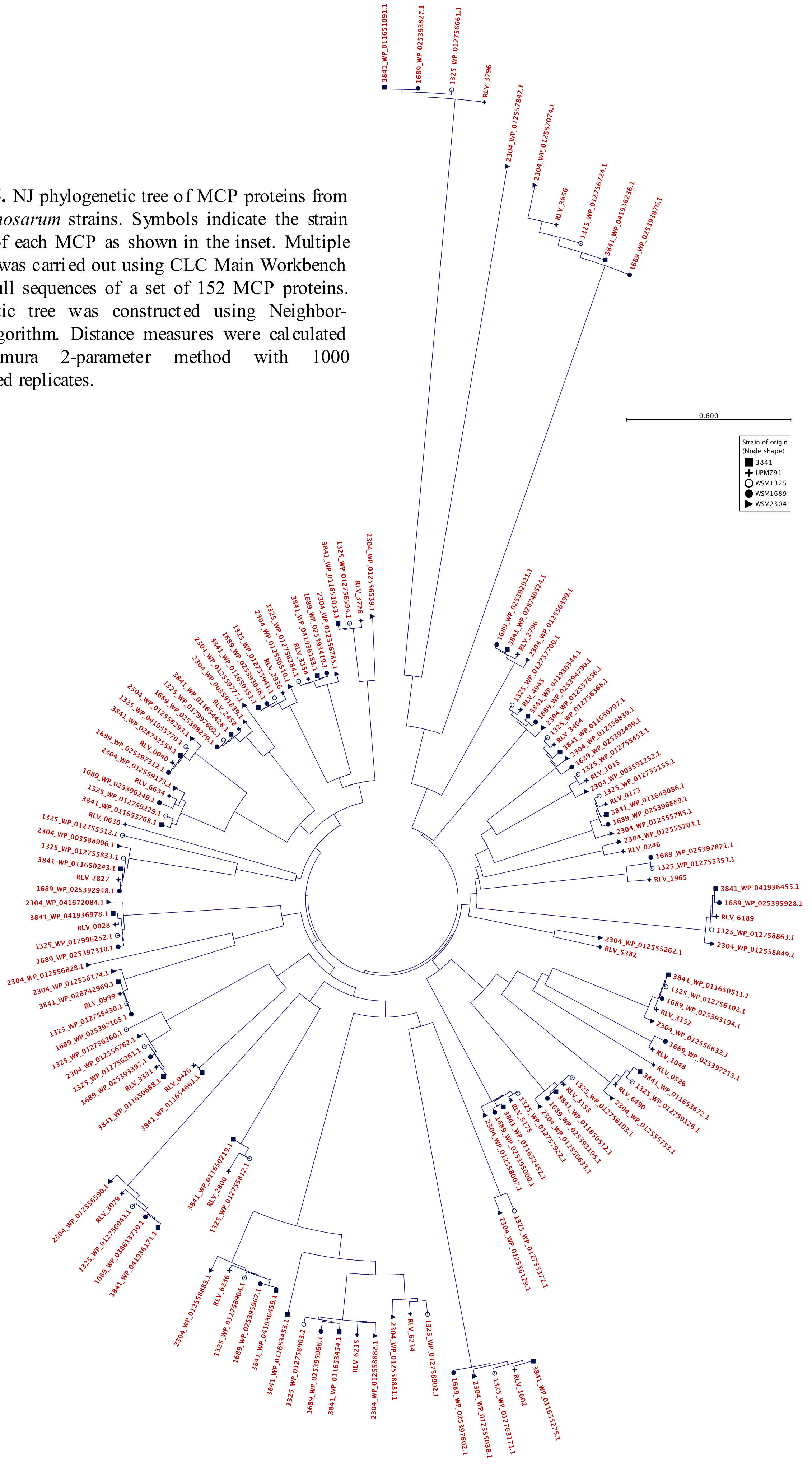

Supplement: Supplementary file 1 [file genes-09-00060-s001.zip › Sanchez-Canizares et al UPM791 genome Supplementary Figures/Figure_S5.pdf]
