# Supplementary material for: Genomic Diversity in the Endosymbiotic Bacterium Rhizobium leguminosarum
Source: Genes (Basel). 2018 Jan 24;9(2):60. doi: 10.3390/genes9020060 (PMC5852556; doi:10.3390/genes9020060)
Supplement: Supplementary file 1 [file genes-09-00060-s001.zip › Sanchez-Canizares et al UPM791 genome Supplementary Figures/Supplementary Table 1.pdf]

	Total		Chromosome		pRlvA		pRlvB		pRlvE		pRlvC		pRlvD	
**TIGRfam category**	**No Prots**	%	**No Prots**	%	**No Prots**	%	**No Prots**	%	**No Prots**	%	**No Prots**	%	**No Prots**	%
Amino acid biosynthesis	90	1.23	81	1.77	4	0.35	1	0.19	1	0.19	2	0.60	1	0.49
Biosynthesis of cofactors, prosthetic groups, and carriers	133	1.82	97	2.12	9	0.78	14	2.65	2	0.39	11	3.27	0	0.00
Cell envelope	254	3.47	199	4.35	21	1.82	15	2.84	12	2.33	8	2.38	2	0.97
Cellular processes	215	2.94	150	3.28	18	1.56	13	2.46	10	1.95	14	4.17	11	5.34
Central intermediary metabolism	69	0.94	44	0.96	6	0.52	1	0.19	6	1.17	17	5.06	0	0.00
DNA metabolism	134	1.83	95	2.08	18	1.56	5	0.95	5	0.97	8	2.38	3	1.46
Energy metabolism	343	4.69	223	4.87	55	4.76	37	6.99	12	2.33	13	3.87	3	1.46
Fatty acid and phospholipid metabolism	56	0.77	39	0.85	4	0.35	3	0.57	9	1.75	1	0.30	0	0.00
Hypothetical proteins	1438	19.66	932	20.36	209	18.08	111	20.98	59	11.48	76	22.62	57	27.67
Mobile and extrachromosomal element functions	123	1.68	22	0.48	20	1.73	7	1.32	8	1.56	19	5.65	47	22.82
Protein fate	245	3.35	180	3.93	17	1.47	14	2.65	13	2.53	18	5.36	2	0.97
Protein synthesis	145	1.98	139	3.04	3	0.26	1	0.19	2	0.39	0	0.00	0	0.00
Purines, pyrimidines, nucleosides and nucleotides	62	0.85	59	1.29	1	0.09	1	0.19	0	0.00	1	0.30	0	0.00
Regulatory functions	408	5.58	258	5.64	81	7.01	29	5.48	24	4.67	12	3.57	3	1.46
Signal transduction	97	1.33	67	1.46	14	1.21	10	1.89	3	0.58	1	0.30	2	0.97
Transcription	76	1.04	54	1.18	7	0.61	8	1.51	2	0.39	5	1.49		0.00
Transport and binding proteins	906	12.39	476	10.40	183	15.83	91	17.20	128	24.90	27	8.04	4	1.94
Unknown function	2057	28.13	1194	26.09	383	33.13	148	27.98	178	34.63	82	24.40	58	28.16
unclassified	663	9.07	398	8.70	123	10.64	35	6.62	49	9.53	40	11.90	16	7.77

No Prots = Number of Proteins.
