# Supplementary figures and images for: Genomic Diversity in the Endosymbiotic Bacterium Rhizobium leguminosarum
Source: Genes (Basel). 2018 Jan 24;9(2):60. doi: 10.3390/genes9020060 (PMC5852556; doi:10.3390/genes9020060)

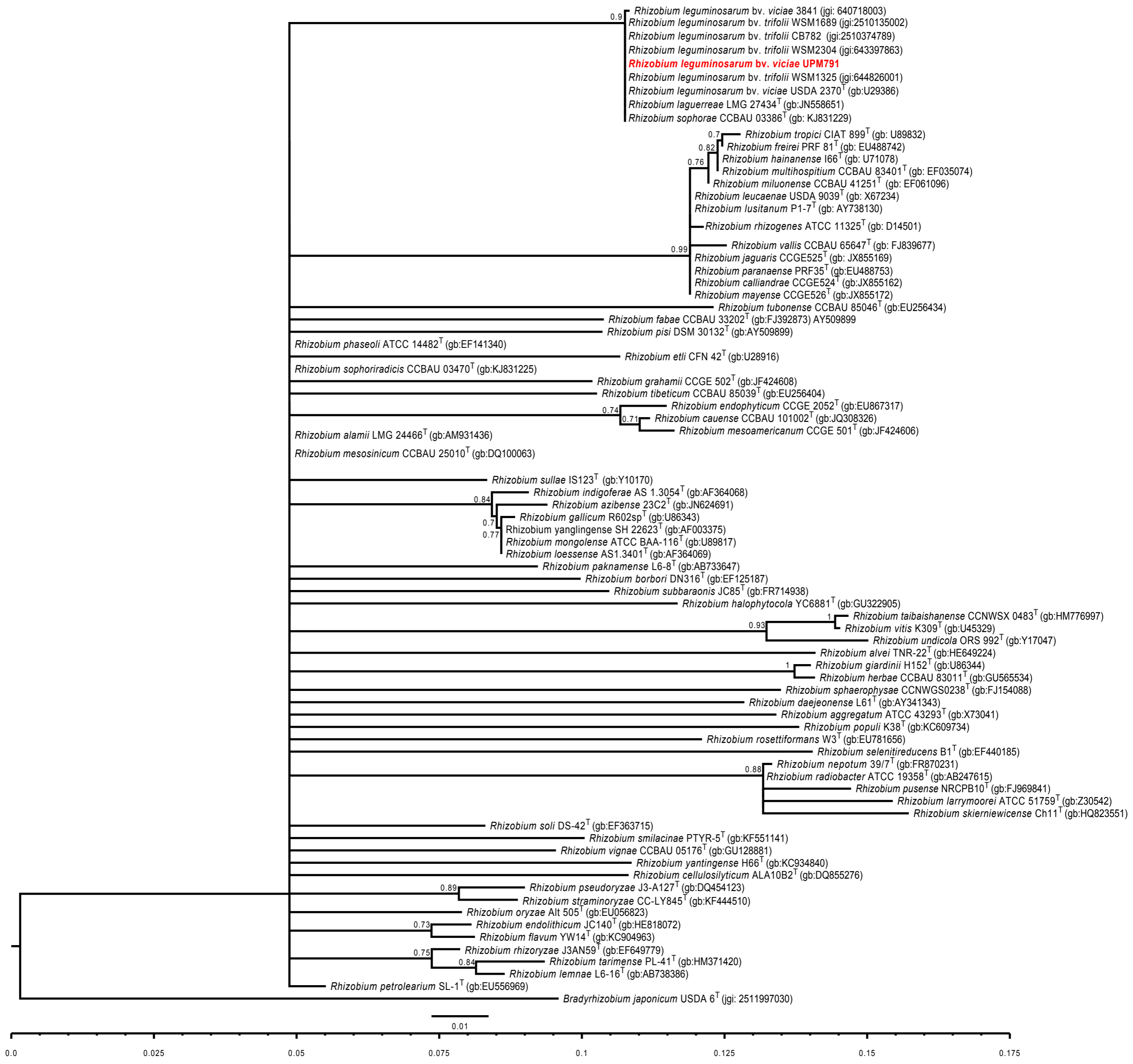

Supplement: Supplementary file 1 [file genes-09-00060-s001.zip › Sanchez-Canizares et al UPM791 genome Supplementary Figures/Figure_S1.pdf]

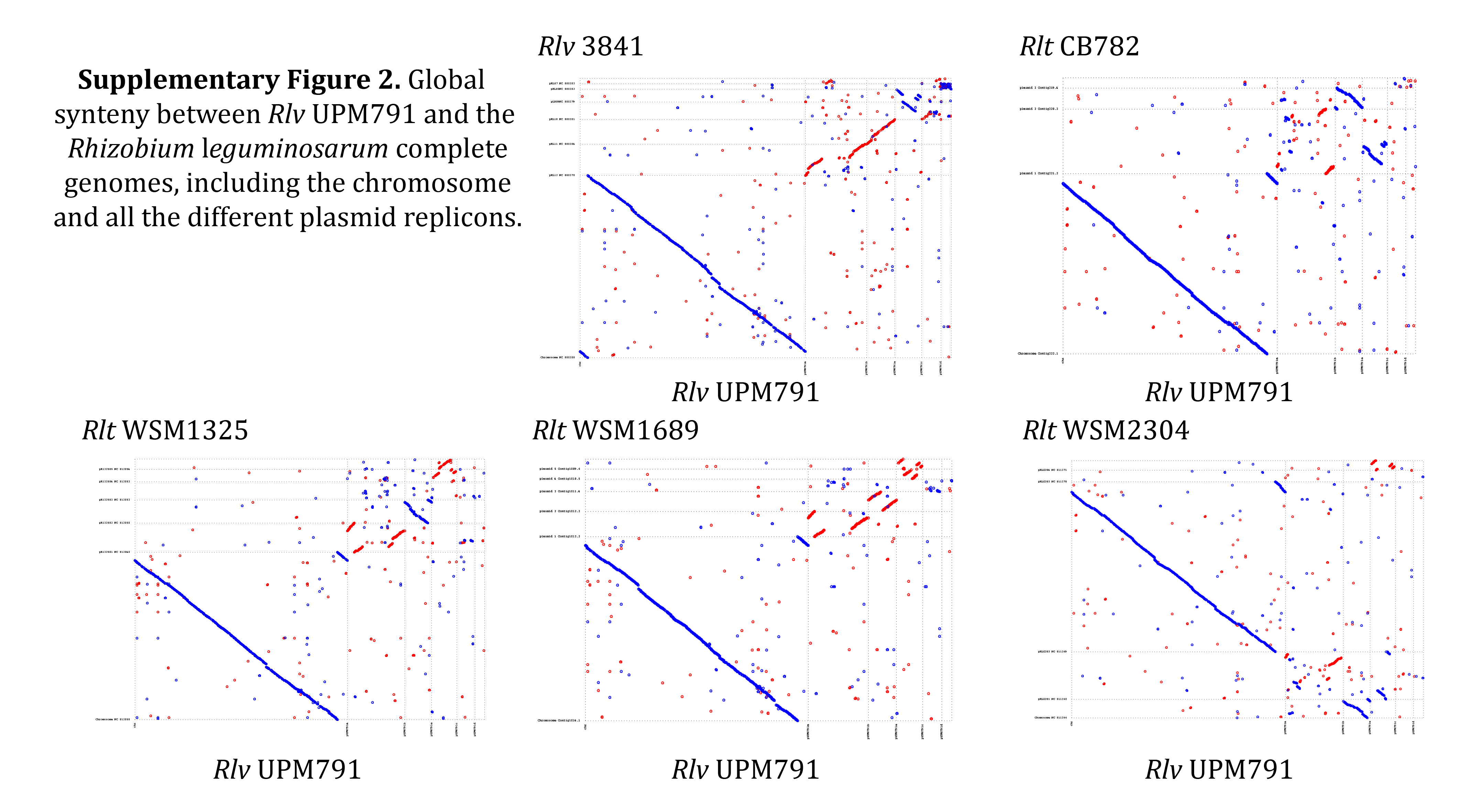

Supplement: Supplementary file 1 [file genes-09-00060-s001.zip › Sanchez-Canizares et al UPM791 genome Supplementary Figures/Figure_S2.jpg]

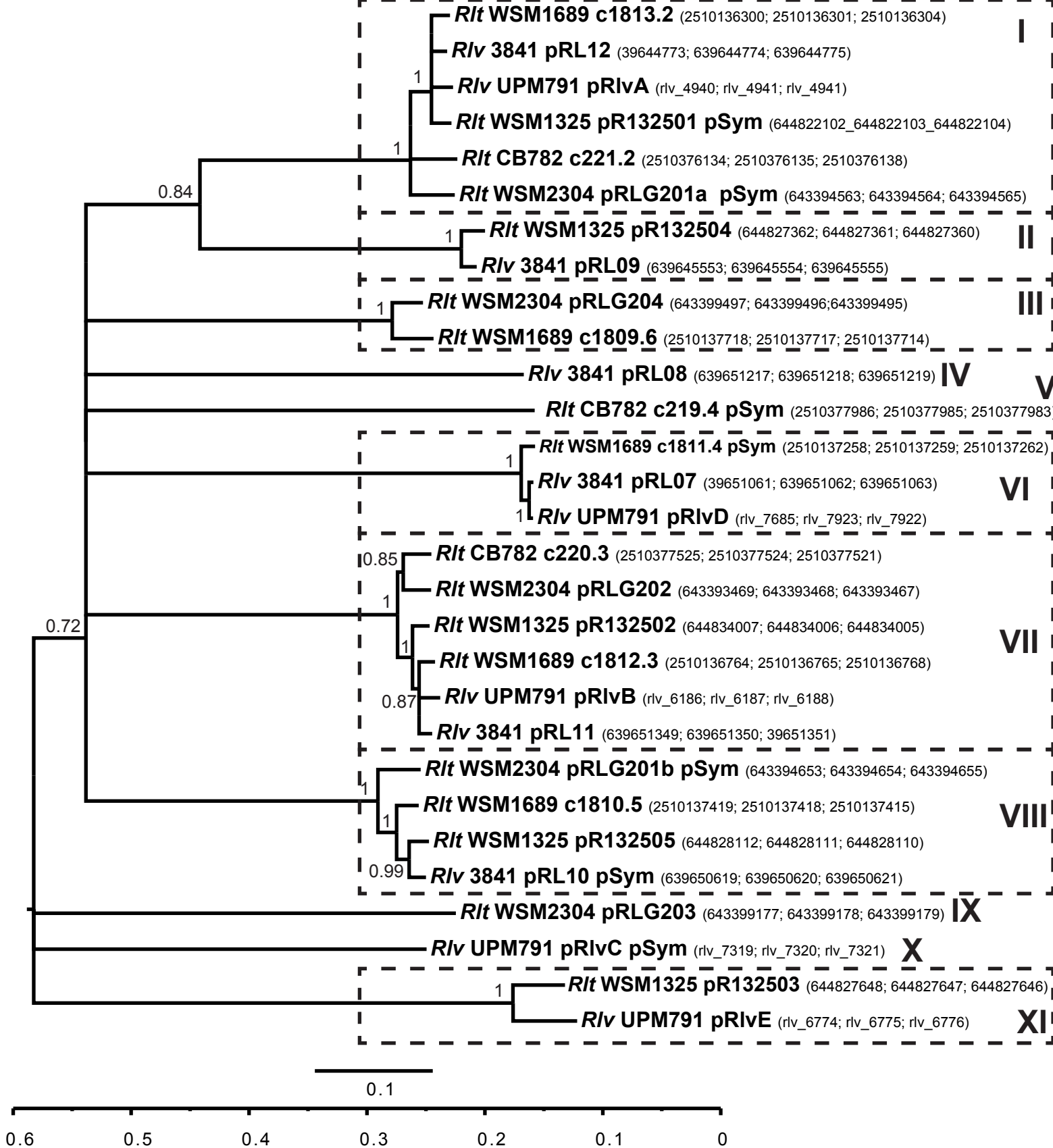

Supplement: Supplementary file 1 [file genes-09-00060-s001.zip › Sanchez-Canizares et al UPM791 genome Supplementary Figures/Figure_S3_.pdf]

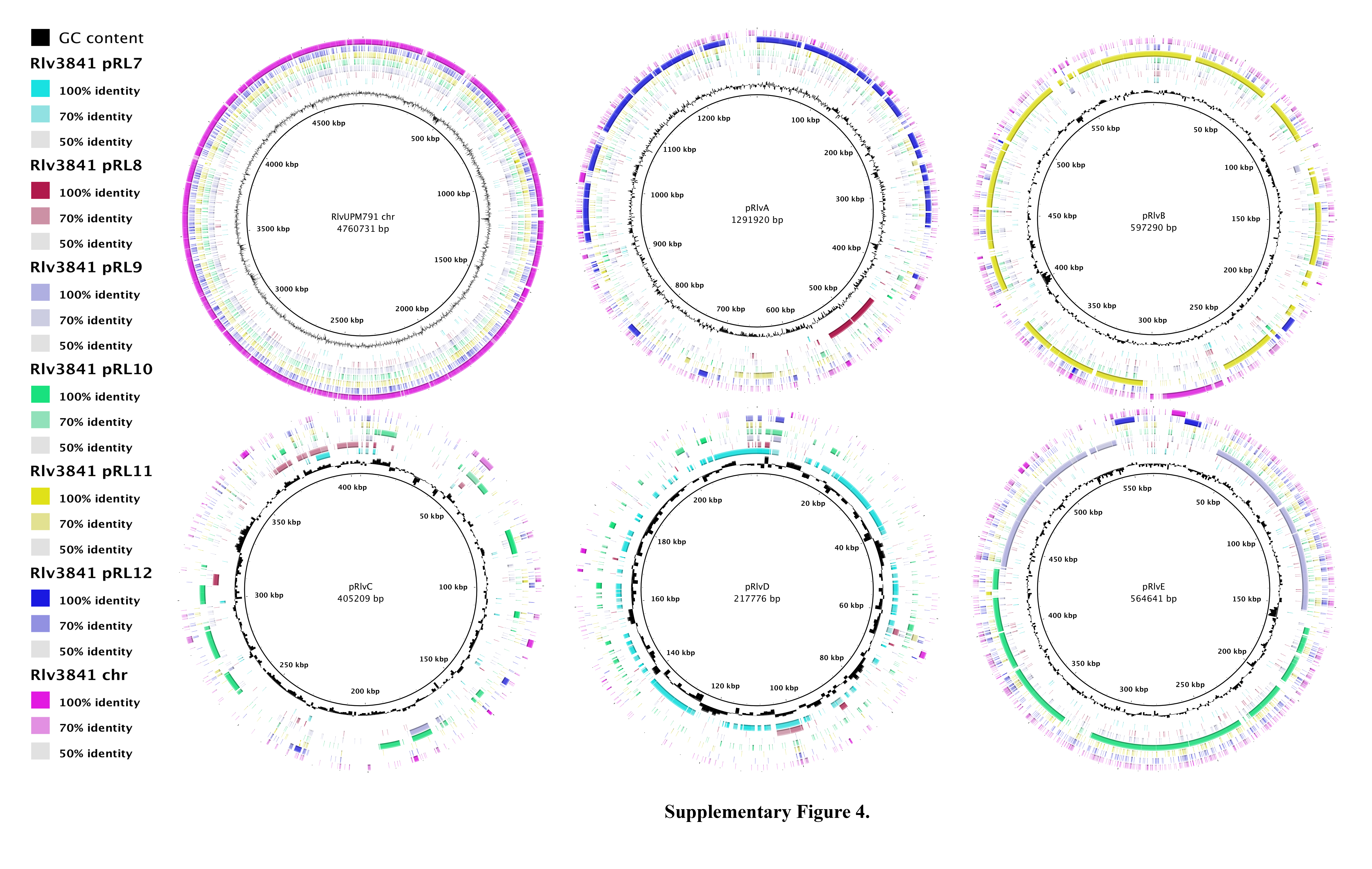

Supplement: Supplementary file 1 [file genes-09-00060-s001.zip › Sanchez-Canizares et al UPM791 genome Supplementary Figures/Figure_S4.jpg]
